# Global practice variation in pharmacologicthromboprophylaxis for general and gynaecologicalsurgery: systematic review

**DOI:** 10.1093/bjsopen/zrac129

**Published:** 2022-10-07

**Authors:** Negar Pourjamal, Lauri I Lavikainen, Alex L E Halme, Rufus Cartwright, Kaisa Ahopelto, Gordon H Guyatt, Kari A O Tikkinen

**Affiliations:** Faculty of Medicine, University of Helsinki, Helsinki, Finland; Faculty of Medicine, University of Helsinki, Helsinki, Finland; Faculty of Medicine, University of Helsinki, Helsinki, Finland; Department of Obstetrics and Gynaecology, London North West Healthcare NHS Trust, London, UK; Department of Epidemiology & Biostatistics, Imperial College London, London, UK; Department of Transplantation and Liver Surgery, University of Helsinki and Helsinki University Hospital, Helsinki, Finland; Department of Health Research Methods, Evidence and Impact, McMaster University, Hamilton, Canada; Department of Medicine, McMaster University, Hamilton, Canada; Department of Urology, University of Helsinki and Helsinki University Hospital, Helsinki, Finland; Department of Surgery, South Karelian Central Hospital, Lappeenranta, Finland

*Dear Editor*

Venous thromboembolism (VTE) and bleeding are serious complications of surgery. Pharmacological prophylaxis decreases VTE but increases bleeding^1^. The decision to use thromboprophylaxis requires balancing decreasing VTE *versus* increased risk of bleeding^2^. Expert recommendations regarding VTE prophylaxis in surgery vary^3^, but the extent of practice variation remains uncertain.

We performed comprehensive literature searches in Embase, MEDLINE, Web of Science, and Google Scholar for observational studies with procedure-specific information on VTE and/or bleeding for 16 general abdominal and 22 gynaecological surgery procedures until November 2020^3^ (PROSPERO CRD42021234119) (*Supplementary material*). Two reviewers independently assessed eligibility and extracted data using standardized, piloted data forms, guided by written instructions. We included surgical procedures that had been investigated in at least five studies, with the majority of participants enrolled from 2000 onwards (*Supplementary material*). For each study, we extracted data on the proportion of patients receiving pharmacological prophylaxis, and the duration of prophylaxis. For each procedure, we calculated the proportion of discretionary use of pharmacological prophylaxis, and the mean or median duration of prophylaxis.

Of 32 523 potentially relevant titles and abstracts, 4082 warranted full-text review, of which 50 reports addressing five general and two gynaecological procedures were eligible: laparoscopic cholecystectomy (7 studies), open gastric bypass (7), open groin hernia repair (6), open liver resection (6), open ovarian cancer surgery (9), open radical hysterectomy (5), and laparoscopic sleeve gastrectomy (10) (*Figs. S1* and *S2* and *Table S1*). *Table S2* presents study characteristics.

All 50 studies reported whether prophylaxis was used; 29 of 46 (63 per cent) studies in which prophylaxis was used, also reported the duration of use.

Studies consistently reported high rates of prophylaxis in open gastric bypass, laparoscopic sleeve gastrectomy, open radical hysterectomy (one exception), and open ovarian cancer surgery (one exception). The duration of pharmacological prophylaxis varied between 4 and 20 days after open gastric bypass, between 1 and 35 days after laparoscopic sleeve gastrectomy, between 4 and 12 days after open radical hysterectomy, and between 7 and 28 days after open ovarian cancer surgery (*Fig. 1*).

**Fig. 1:**
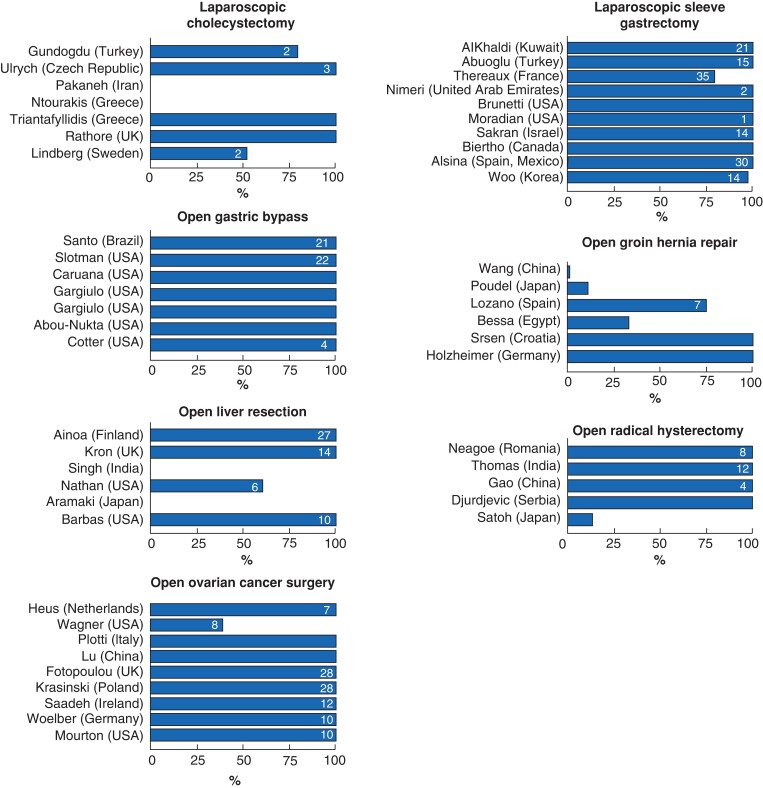
Proportion (in percentages) of patients with reported use of pharmacological prophylaxis Depending on the study, the mean or median duration of pharmacological prophylaxis (in days) for those who received any is noted on each bar (if reported in the article).

The proportion receiving prophylaxis varied widely in laparoscopic cholecystectomy, open groin hernia repair, and open liver resection. The duration of pharmacological prophylaxis varied between 2 and 3 days after laparoscopic cholecystectomy, between 6 and 27 days after open liver resection, and only one study reported the duration for hernia repair (*Fig. 1*).

Studies of laparoscopic cholecystectomy, open hernia repair, and open liver resection reported large variation in the use of pharmacological prophylaxis (*Fig. 1*). Studies in cancer and obesity surgery consistently reported high rates of use of pharmacological prophylaxis, but with substantial variation in the duration of prophylaxis.

Earlier studies have addressed pharmacological prophylaxis in one or more centres^4,5^, but our study examines variation in practice across studies. The other strengths of this review include a thorough search of contemporary studies, application of explicit eligibility criteria, and standardized piloted data forms for data collection.

This article has limitations. Although we screened many potential studies, only a small proportion proved eligible (1.4 per cent of full texts screened). These 50 studies (of which 15 had low risk of bias, 19 had moderate risk, and 16 had high risk of bias; *Tables S3* and *S4*) published between 2000 and 2020 represent seven procedures each with 5–10 studies.

We identified substantial practice variation, within and between countries, in the use of pharmacological prophylaxis in most types of benign surgery, and in the duration of prophylaxis after cancer surgery. Rationalization of practice will require evidence that provides a better understanding of procedure-specific risks of VTE and bleeding as well as creation of procedure-specific, evidence-based guidelines for thromboprophylaxis. Rationalization of current practice would decrease both under- and overuse of thromboprophylaxis improving patient outcomes.

## Supplementary Material

zrac129_Supplementary_Data

## Data Availability

The corresponding author is the custodian of the data and will provide access to data on request.

## References

[1] Marcucci M, Etxeandia-Ikobaltzeta I, Yang S, Germini F, Gupta S, Agarwal A et al Benefits and harms of direct oral anticoagulation and low molecular weight heparin for thromboprophylaxis in patients undergoing noncardiac surgery: systematic review and network meta-analysis of randomised trials. BMJ 2022;376:e066785 10.1136/bmj-2021-066785 PMC8905353 35264372

[2] Tikkinen KAO, Guyatt GH. Baseline risks of venous thromboembolism and major bleeding are crucial in decision-making on thromboprophylaxis. Eur Urol 2020;78:369–370 32536485 10.1016/j.eururo.2020.05.032

[3] Lavikainen LI, Guyatt GH, Lee Y, Couban RJ, Luomaranta AL, Sallinen VJ et al Systematic reviews of observational studies of Risk of Thrombosis and Bleeding in General and Gynecologic Surgery (ROTBIGGS): introduction and methodology. Syst Rev 2021;10:264 34625092 10.1186/s13643-021-01814-2 PMC8499502

[4] Krell RW, Scally CP, Wong SL, Abdelsattar ZM, Birkmeyer NJO, Fegan K, et al Variation in hospital thromboprophylaxis practices for abdominal cancer surgery. Ann Surg Oncol 2016;23:1431–1439 26567148 10.1245/s10434-015-4970-9

[5] Weiss MJ, Kim Y, Ejaz A, Spolverato G, Haut ER, Hirose K et al Venous thromboembolic prophylaxis after a hepatic resection: patterns of care among liver surgeons. HPB (Oxford) 2014;16:892–898 24888461 10.1111/hpb.12278 PMC4238855

