# Supplementary material for: Global practice variation in pharmacologicthromboprophylaxis for general and gynaecologicalsurgery: systematic review
Source: BJS Open. 2022 Oct 7;6(5):zrac129. doi: 10.1093/bjsopen/zrac129 (PMC9539740; doi:10.1093/bjsopen/zrac129)
Supplement: zrac129_Supplementary_Data [file zrac129_supplementary_data.docx]

**Global practice variation in pharmacologic thromboprophylaxis for general and gynecological surgery: Results from a systematic analysis**

Negar Pourjamal^1†^, Lauri I. Lavikainen^1†^, Alex L. E. Halme^1^, Rufus Cartwright^2,3^, Kaisa Ahopelto^4^, Gordon H. Guyatt^5,6^, Kari A. O. Tikkinen^7,8^

1. Faculty of Medicine, University of Helsinki, Helsinki, Finland
2. Department of Obstetrics and Gynaecology, LNWH NHS Trust, London, UK
3. Department of Epidemiology & Biostatistics, Imperial College London, UK
4. Department of Transplantation and Liver Surgery, University of Helsinki and Helsinki University Hospital, Helsinki, Finland
5. Department of Health Research Methods, Evidence and Impact, McMaster University, Hamilton, ON, Canada
6. Department of Medicine, McMaster University, Hamilton, ON, Canada
7. Department of Urology, University of Helsinki and Helsinki University Hospital, Helsinki, Finland
8. Department of Surgery, South Karelian Central Hospital, Lappeenranta, Finland

† Shared first authorship.

**Corresponding author.** Kari A. O. Tikkinen. Department of Urology, Helsinki University Hospital, Haartmaninkatu 4, 00029 Helsinki, Finland. E-mail address: [kari.tikkinen@helsinki.fi](mailto:kari.tikkinen@helsinki.fi). **ORCID ID:** 0000-0002-1389-8214; **Twitter:** @KariTikkinen

**Supplementary Materials - Index**

| **Supplementary Methods** |  |
| --- | --- |
| 1. Search history for baseline risk of VTE and major bleeding in general surgery | *pag. 3-13* |
| 2. Search history for baseline risk of VTE and major bleeding in gynecological surgery  3. Further details regarding study selection | *pag. 14-26*  *pag. 27* |
| **Supplementary Appendix** |  |
| Appendix S1. The Risk of Thrombosis and Bleeding in General and Gynecologic Surgery (ROTBIGGS) Investigators | *pag. 28* |
| **Supplementary Figures and Tables** |  |
| Figure S1. Study flow chart (general surgery) | *pag. 29* |
| Figure S2. Study flow chart (gynecological surgery)  Table S1. List of the included 50 studies, stratified by procedure (in chronological order)  Table S2. Summary of included studies by procedure  Table S3. Design features used for assessment of risk of bias  Table S4. Risk of bias in individual studies | *pag. 30*  *pag. 31-35*  *pag. 36*  *pag. 37*  *pag. 38-39* |
|  |  |

##

## **Supplementary Methods**

## **1. Search history for baseline risk of VTE and major bleeding in general surgery**

Database: OVID Medline Epub Ahead of Print, In-Process & Other Non-Indexed Citations, Ovid MEDLINE(R) Daily and Ovid MEDLINE(R) 1946 to March 15, 2019

Search Strategy:

--------------------------------------------------------------------------------

((chemoprophylax* or chemoprophylactic* or prophylax* or prophylactic*) and (venous or vein or thromb*)).ti,ab.

(prevent* adj3 (venous or vein or thromb*)).mp.

(thromboprophylax* or thromboprophylactic*).mp.

*Postoperative Complications/

Postoperative Complications/ep, et, pc

Risk Factors/

(ep or ae).fs. and (venous or thromb* or bleed* or haemorr* or hemorr*).ti,ab.

(risk* or high-risk or incidence* or meta?analysis or analysis or complication* or outcome* or safety or versus or thrombosis or transfusion* or adverse or bleed* or haemorr* or hemorr*).ti.

or/1-8

embolism/ or exp pulmonary embolism/ or exp thromboembolism/

exp Thrombosis/

(DVT or VTE or PE).ti,ab.

((venous or vein or pulmonary or lung) adj3 (emboli* or thromb*)).mp.

(DVT or VTE or PE or PTE).ti,ab.

or/10-14

9 and 15

Appendectomy/ or exp Bariatric Surgery/ or exp Cholecystectomy/ or exp Colectomy/ or exp Gastrectomy/ or Hepatectomy/ or Herniorrhaphy/ or pancreatectomy/ or Pancreaticoduodenectomy/ or pancreaticojejunostomy/ or Splenectomy/

General Surgery/ or exp digestive system surgical procedures/

exp Digestive System/su or Cholecystitis/su or Gallbladder/su or exp Gallbladder Diseases/su or Hernia, Abdominal/su or Hernia, Inguinal/su or exp Hernia, Ventral/su or exp Intestinal Diseases/su or exp Liver Diseases/su or exp Pancreas/su or exp Pancreatic Diseases/su or Spleen/su or exp Splenic Diseases/su or exp Stomach Diseases/su

(appendectom* or appendicectom* or colectomy* or proctocolectom* or cholecystectom* or duodenectom* or gastrectom* or hernioplast* or herniorrhaph* or herniotom* or jejunectom* or pancreatectom* or pancreaticojejunostom* or pancreaticoduodenectom* or duodenopancreatectom*).mp.

((surgery or resection* or excision* or repair* or operation* or laproscop* or laparoscop* or sleeve*) adj3 (abdominoperineal or perineal or anal* or anus or appendix or bowel* or colon* or duoden* or jejun* or ileal* or ileum* or jejuno?ileal or intestine* or gall bladder or gall?bladder or gastric or bariatric* or stomach or hernia or liver or adenoma or hepatoma* or hepatocellular* or rectal* or rectum)).mp.

((general or abdominal or major) adj3 (surgery or surgical)).mp.

(prolapse adj3 rectal).mp.

(Rectopexy or rectosigmoidectom* or sigmoidectom* or DHoore or d'hoore or Delorme or Altemeier).mp.

or/17-24

16 and 25

9 and 15 and 25

**EMBASE**

Database: Embase <1974 to 2019 March 11>

Search Strategy:

--------------------------------------------------------------------------------

((chemoprophylax* or chemoprophylactic* or prophylax* or prophylactic*) and (venous or vein or thromb*)).ti,ab. (28841)

(prevent* adj3 (venous or vein or thromb*)).mp. (28252)

(thromboprophylax* or thromboprophylactic*).mp. (7355)

*postoperative complication/co, ep, et, pc [Complication, Epidemiology, Etiology, Prevention] (33531)

exp *venous thromboembolism/co, ep, et, pc [Complication, Epidemiology, Etiology, Prevention] (18251)

thrombosis prevention/ (10458)

postoperative complication/ep [Epidemiology] (9157)

*venous thromboembolism/ (14535)

*deep vein thrombosis/ (15764)

venous thromboembolism/ep [Epidemiology] (1367)

risk factor/ (925628)

(ep or co).fs. and (venous or thromb* or bleed* or haemorr* or hemorr*).ti,ab.

(220502)

(risk* or high-risk or incidence* or meta?analysis or analysis or complication* or outcome* or safety or versus or thrombosis or transfusion* or adverse or bleed* or haemorr* or hemorr*).ti. (2707778)

or/1-13 (3486260)

Annotation: post op VTE comp

exp thromboembolism/ (440725)

(DVT or VTE or PE or PTE).ti,ab. (82850)

((venous or vein or pulmonary or lung) adj3 (emboli* or thromb*)).mp. (216179)

or/15-17 (506092)

Annotation: VTE broad

14 and 18 (224773)

Annotation: risk of post-op VTE

general surgery/ (13528)

exp abdominal surgery/ (708663)

exp gastrointestinal surgery/ (321622)

cholecystitis/su [Surgery] (3221)

gallbladder disease/su [Surgery] (1698)

exp abdominal wall hernia/su [Surgery] (15449)

exp enteropathy/su [Surgery] (124538)

exp enteropathy/su [Surgery] (124538)

exp liver disease/su [Surgery] (61513)

exp pancreas disease/su [Surgery] (33926)

exp spleen disease/su [Surgery] (6989)

exp stomach disease/su [Surgery] (42619)

((general or abdominal or major) adj3 (surgery or surgical)).mp. (102145)

(prolapse adj3 rectal).mp. [mp=title, abstract, heading word, drug trade name, original title, device manufacturer, drug manufacturer, device trade name, keyword, floating subheading word, candidate term word] (3266)

(rectopexy or proctopexy or rectosigmoidectom* or sigmoidectom* or DHoore or d'hoore or Delorme or Altemeier).mp. (5621)

(surgery or resection* or excision* or repair* or operation* or laproscop* or sleeve*).mp. and (exp digestive system/ or exp spleen/) [mp=title, abstract, heading word, drug trade name, original title, device manufacturer, drug manufacturer, device trade name, keyword, floating subheading word, candidate term word] (233115)

(appendectom* or appendicectom* or colectomy* or proctocolectom* or cholecystectom* or duodenectom* or gastrectom* or hernioplast* or herniorrhaph* or herniotom* or jejunectom* or pancreatectom* or pancreaticojejunostom* or pancreaticoduodenectom* or duodenopancreatectom*).mp. (189962)

((surgery or resection* or excision* or repair* or operation* or laproscop* or laparoscop* or sleeve*) adj3 (abdominoperineal or anal* or anus or appendix or bowel* or colon* or duoden* or jejun* or ileal* or ileum* or jejuno?ileal or intestine* or gall bladder or gall?bladder or gastric or bariatric* or stomach or hernia or liver or adenoma or hepatoma* or hepatocellular* or rectal* or rectum)).mp. (273066)

or/20-37 (1022761)

14 and 18 and 38 (22794)

exp animals/ or exp invertebrate/ or animal experiment/ or animal model/ or animal tissue/ or animal cell/ or nonhuman/ (25472734)

human/ or normal human/ or human cell/ (19402712)

40 and 41 (19349027)

40 not 42 (6123707)

39 not 43 (22544)

exp controlled clinical trial/ (718277)

44 not 45 (21607)

clinical study/ (151683)

case control study/ (136785)

family study/ (25001)

longitudinal study/ (121983)

retrospective study/ (740307)

prospective study/ (500661)

cohort analysis/ (442572)

(Cohort adj (study or studies)).mp. (251414)

(Case control adj (study or studies)).tw. (119867)

(follow up adj (study or studies)).tw. (58795)

(observational adj (study or studies)).tw. (139036)

(epidemiologic$ adj (study or studies)).tw. (98776)

(cross sectional adj (study or studies)).tw. (180909)

or/47-59 (2283762)

46 and 60 (5348)

(prognosis or prognostic or predict* or risk*).mp. [mp=title, abstract, heading word, drug trade name, original title, device manufacturer, drug manufacturer, device trade name, keyword, floating subheading word, candidate term word] (5487179)

(incidence* or outcome* or comparison* or complication*).ti. (1083287)

prevalence.mp. or prevalence/ (939978)

baseline.mp. (829438)

or/62-65 (7111926)

46 and 66 (14542)

61 or 67 (15593)

transplant*.ti,kw,jw. (479040)

transplant*.ab. /freq=2 (315144)

69 or 70 (555136)

68 not 71 (12241)

🡪73 remove 209 duplicates in Endnote (12032)

🡪74 limit 73 to year =>2004 in Endnote (10467)

**Web of Science**

🡪#20 7304 remove duplicates in endnote

# 19 **[7,323](http://apps.webofknowledge.com.libaccess.lib.mcmaster.ca/summary.do?product=WOS&doc=1&qid=131&SID=7ERKFWRD7WVwZpcUw6x&search_mode=AdvancedSearch&update_back2search_link_param=yes)** #17 NOT #18

*Indexes=SCI-EXPANDED, SSCI, A&HCI, CPCI-S, CPCI-SSH, ESCI Timespan=2004-2019*

# 18 **[410,495](http://apps.webofknowledge.com.libaccess.lib.mcmaster.ca/summary.do?product=WOS&doc=1&qid=79&SID=7ERKFWRD7WVwZpcUw6x&search_mode=AdvancedSearch&update_back2search_link_param=yes)** TS=transplant*

*Indexes=SCI-EXPANDED, SSCI, A&HCI, CPCI-S, CPCI-SSH, ESCI Timespan=2004-2019*

# 17 **[8,098](http://apps.webofknowledge.com.libaccess.lib.mcmaster.ca/summary.do?product=WOS&doc=1&qid=130&SID=7ERKFWRD7WVwZpcUw6x&search_mode=AdvancedSearch&update_back2search_link_param=yes)** #16

*Indexes=SCI-EXPANDED, SSCI, A&HCI, CPCI-S, CPCI-SSH, ESCI Timespan=2004-2019*

# 16 **[8,098](http://apps.webofknowledge.com.libaccess.lib.mcmaster.ca/summary.do?product=WOS&doc=1&qid=129&SID=7ERKFWRD7WVwZpcUw6x&search_mode=CombineSearches&update_back2search_link_param=yes)** #15 AND #14

*Indexes=SCI-EXPANDED, SSCI, A&HCI, CPCI-S, CPCI-SSH, ESCI Timespan=2004-2019*

# 15 **[11,374,071](http://apps.webofknowledge.com.libaccess.lib.mcmaster.ca/summary.do?product=WOS&doc=1&qid=76&SID=7ERKFWRD7WVwZpcUw6x&search_mode=AdvancedSearch&update_back2search_link_param=yes)** TS=(cohort or observational or cross-sectional or longitudinal NEAR/2 study or studies)

*Indexes=SCI-EXPANDED, SSCI, A&HCI, CPCI-S, CPCI-SSH, ESCI Timespan=2004-2019*

# 14 **[20,183](http://apps.webofknowledge.com.libaccess.lib.mcmaster.ca/summary.do?product=WOS&doc=1&qid=128&SID=7ERKFWRD7WVwZpcUw6x&search_mode=CombineSearches&update_back2search_link_param=yes)** #13 AND #8

*Indexes=SCI-EXPANDED, SSCI, A&HCI, CPCI-S, CPCI-SSH, ESCI Timespan=2004-2019*

# 13 **[1,171,802](http://apps.webofknowledge.com.libaccess.lib.mcmaster.ca/summary.do?product=WOS&doc=1&qid=104&SID=7ERKFWRD7WVwZpcUw6x&search_mode=CombineSearches&update_back2search_link_param=yes)** #12 OR #11 OR #10 OR #9

*Indexes=SCI-EXPANDED, SSCI, A&HCI, CPCI-S, CPCI-SSH, ESCI Timespan=2004-2019*

# 12 **[875,276](http://apps.webofknowledge.com.libaccess.lib.mcmaster.ca/summary.do?product=WOS&doc=1&qid=103&SID=7ERKFWRD7WVwZpcUw6x&search_mode=AdvancedSearch&update_back2search_link_param=yes)** TI=(complication* or outcome* or safety or versus or thrombosis or transfusion* or adverse or bleed* or haemorr* or hemorr*)

*Indexes=SCI-EXPANDED, SSCI, A&HCI, CPCI-S, CPCI-SSH, ESCI Timespan=2004-2019*

# 11 **[4,861](http://apps.webofknowledge.com.libaccess.lib.mcmaster.ca/summary.do?product=WOS&doc=1&qid=72&SID=7ERKFWRD7WVwZpcUw6x&search_mode=AdvancedSearch&update_back2search_link_param=yes)** TS=(thromboprophylax* or thromboprophylactic*)

*Indexes=SCI-EXPANDED, SSCI, A&HCI, CPCI-S, CPCI-SSH, ESCI Timespan=2004-2019*

# 10 **[358,947](http://apps.webofknowledge.com.libaccess.lib.mcmaster.ca/summary.do?product=WOS&doc=1&qid=71&SID=7ERKFWRD7WVwZpcUw6x&search_mode=AdvancedSearch&update_back2search_link_param=yes)** TS=(prevent* NEAR/3 venous or vein or thromb*)

*Indexes=SCI-EXPANDED, SSCI, A&HCI, CPCI-S, CPCI-SSH, ESCI Timespan=2004-2019*

# 9 **[4,145](http://apps.webofknowledge.com.libaccess.lib.mcmaster.ca/summary.do?product=WOS&doc=1&qid=70&SID=7ERKFWRD7WVwZpcUw6x&search_mode=AdvancedSearch&update_back2search_link_param=yes)** TS=((chemoprophylax* or chemoprophylactic*or prophylax* or prophylactic*) and (venous or vein or thromb*))

*Indexes=SCI-EXPANDED, SSCI, A&HCI, CPCI-S, CPCI-SSH, ESCI Timespan=2004-2019*

# 8 **[31,477](http://apps.webofknowledge.com.libaccess.lib.mcmaster.ca/summary.do?product=WOS&doc=1&qid=127&SID=7ERKFWRD7WVwZpcUw6x&search_mode=CombineSearches&update_back2search_link_param=yes)** #7 AND #4

*Indexes=SCI-EXPANDED, SSCI, A&HCI, CPCI-S, CPCI-SSH, ESCI Timespan=2004-2019*

# 7 **[282,903](http://apps.webofknowledge.com.libaccess.lib.mcmaster.ca/summary.do?product=WOS&doc=1&qid=126&SID=7ERKFWRD7WVwZpcUw6x&search_mode=CombineSearches&update_back2search_link_param=yes)** #6 OR #5

*Indexes=SCI-EXPANDED, SSCI, A&HCI, CPCI-S, CPCI-SSH, ESCI Timespan=2004-2019*

# 6 **[271,002](http://apps.webofknowledge.com.libaccess.lib.mcmaster.ca/summary.do?product=WOS&doc=1&qid=125&SID=7ERKFWRD7WVwZpcUw6x&search_mode=AdvancedSearch&update_back2search_link_param=yes)** TI=(venous or vein or pulmonary or lung NEAR/3 emboli* or thromb*)

*Indexes=SCI-EXPANDED, SSCI, A&HCI, CPCI-S, CPCI-SSH, ESCI Timespan=2004-2019*

# 5 **[22,655](http://apps.webofknowledge.com.libaccess.lib.mcmaster.ca/summary.do?product=WOS&doc=1&qid=66&SID=7ERKFWRD7WVwZpcUw6x&search_mode=AdvancedSearch&update_back2search_link_param=yes)** TS=(DVT or VTE or PE or PTE)

*Indexes=SCI-EXPANDED, SSCI, A&HCI, CPCI-S, CPCI-SSH, ESCI Timespan=2004-2019*

# 4 **[2,747,833](http://apps.webofknowledge.com.libaccess.lib.mcmaster.ca/summary.do?product=WOS&doc=1&qid=117&SID=7ERKFWRD7WVwZpcUw6x&search_mode=CombineSearches&update_back2search_link_param=yes)** #3 OR #2 OR #1

*Indexes=SCI-EXPANDED, SSCI, A&HCI, CPCI-S, CPCI-SSH, ESCI Timespan=2004-2019*

# 3 **[25,450](http://apps.webofknowledge.com.libaccess.lib.mcmaster.ca/summary.do?product=WOS&doc=1&qid=95&SID=7ERKFWRD7WVwZpcUw6x&search_mode=AdvancedSearch&update_back2search_link_param=yes)** TI=(appendectom* or appendicectom* or colectomy* or proctocolectom* or cholecystectom* or duodenectom* or gastrectom* or hernioplast* or herniorrhaph* or herniotom* or jejunectom* or pancreatecom* or pancreaticojejunostom* or pancreaticoduodenectom* or duodenopancreatectom* or rectopexy or rectosigmoidectom* or sigmoidectom* or DHoore or d'hoore or Delorme or Altemeier)

*Indexes=SCI-EXPANDED, SSCI, A&HCI, CPCI-S, CPCI-SSH, ESCI Timespan=2004-2019*

# 2 **[2,541,613](http://apps.webofknowledge.com.libaccess.lib.mcmaster.ca/summary.do?product=WOS&doc=1&qid=116&SID=7ERKFWRD7WVwZpcUw6x&search_mode=AdvancedSearch&update_back2search_link_param=yes)** TI=(surgery or resection* or excision* or repair* or operation* or prolapse* or laproscop* or laparoscop* or sleeve* NEAR/3 abdominoperineal or anal* or anus or appendix or bowel* or colon* or duoden* or jejun* ileal* or ileum* or jejuno?ileal or intestine* or gall bladder or gall?bladder or gastric or bariatric* or stomach or hernia or liver or adenoma or hepatoma* or hepatocellular* or rectal* or rectum)

*Indexes=SCI-EXPANDED, SSCI, A&HCI, CPCI-S, CPCI-SSH, ESCI Timespan=2004-2019*

# 1 **[248,183](http://apps.webofknowledge.com.libaccess.lib.mcmaster.ca/summary.do?product=WOS&doc=1&qid=81&SID=7ERKFWRD7WVwZpcUw6x&search_mode=AdvancedSearch&update_back2search_link_param=yes)** TI= (general or abdominal or major NEAR/3 surgery or surgical)

*Indexes=SCI-EXPANDED, SSCI, A&HCI, CPCI-S, CPCI-SSH, ESCI Timespan=2004-2019*

**Google Scholar**

We queried Google scholar using Harzig’s PublishorPerish version 6.49.6406

<https://harzing.com/resources/publish-or-perish>

We ran two queries (details below) and selected the highest-ranked records for each query (rank=>750) and combined the results in Endnote. NB there was a database error in Q2 and only 980 records were downloaded (instead of 1000)

Query 1

risk, embolism thrombosis DVT VTE PE PTE, general surgery

*Publish or Perish 6.49.6406.7079*

Search terms

**All of the words:** risk
**Any of the words:** embolism thrombosis DVT VTE PE PTE
**The phrase:** general surgery
**Years:** all

Data retrieval

**Data source:** Google Scholar
**Query date:** 28/05/2019 11:21:52 AM
**Cache date:** 28/05/2019 11:44:03 AM
**Query result:** [0] The operation completed successfully.

Metrics

**Reference date:** 28/05/2019 11:21:52 AM
**Publication years:** 1945-2018
**Citation years:** 74 (1945-2019)
**Papers:** 999
**Citations:** 118931
**Citations/year:** 1607.18
**Citations/paper:** 119.05 (*count=188)
**Citations/author:** 39958.01
**Papers/author:** 381.79
**Authors/paper:** 3.46/4.0/4 (mean/median/mode)
**Age-weighed citation rate:** 8904.22 (sqrt=94.36), 2856.27/author
**Hirsch h-index:** 158 (a=4.76, m=2.14, 80933 cites=68.1% coverage)
**Egghe g-index:** 311 (g/h=1.97, 97225 cites=81.7% coverage)
**PoP hI,norm:** 92
**PoP hI,annual:** 1.24

Query 2

thromboembolism incidence, surgery resection excision operation

*Publish or Perish 6.49.6406.7079*

Search terms

**All of the words:** thromboembolism incidence
**Any of the words:** surgery resection excision operation
**Years:** all

Data retrieval

**Data source:** Google Scholar
**Query date:** 28/05/2019 2:01:25 PM
**Cache date:** 28/05/2019 2:23:46 PM
**Query result:** [12152] The server returned an invalid or unrecognized response

Metrics

**Reference date:** 28/05/2019 2:01:25 PM
**Publication years:** 1947-2018
**Citation years:** 72 (1947-2019)
**Papers:** 980
**Citations:** 125726
**Citations/year:** 1746.19
**Citations/paper:** 128.29 (*count=217)
**Citations/author:** 40777.78
**Papers/author:** 331.91
**Authors/paper:** 3.71/4.0/4 (mean/median/mode)
**Age-weighed citation rate:** 8931.95 (sqrt=94.51), 2815.63/author
**Hirsch h-index:** 171 (a=4.30, m=2.38, 82333 cites=65.5% coverage)
**Egghe g-index:** 317 (g/h=1.85, 100935 cites=80.3% coverage)
**PoP hI,norm:** 94
**PoP hI,annual:** 1.31

Search history update searches for baseline risk of VTE and major bleeding in general surgery

**MEDLINE**

Database: OVID Medline Epub Ahead of Print, In-Process & Other Non-Indexed Citations, Ovid MEDLINE(R) Daily and Ovid MEDLINE(R) 1946 to October 27, 2020

Search Strategy:

--------------------------------------------------------------------------------

1 ((chemoprophylax* or chemoprophylactic* or prophylax* or prophylactic*) and (venous or vein or thromb*)).ti,ab. (18812)

2 (prevent* adj3 (venous or vein or thromb*)).mp. (15559)

3 (thromboprophylax* or thromboprophylactic*).mp. [mp=title, abstract, original title, name of substance word, subject heading word, floating sub-heading word, keyword heading word, organism supplementary concept word, protocol supplementary concept word, rare disease supplementary concept word, unique identifier, synonyms] (5164)

4 *Postoperative Complications/ (161247)

5 Postoperative Complications/ep, et, pc [Epidemiology, Etiology, Prevention & Control] (146329)

6 Risk Factors/ (837286)

7 (ep or ae).fs. and (venous or thromb* or bleed* or haemorr* or hemorr*).ti,ab. (209217)

8 (risk* or high-risk or incidence* or meta?analysis or analysis or complication* or outcome* or safety or versus or thrombosis or transfusion* or adverse or bleed* or haemorr* or hemorr*).ti. (2394352)

9 or/1-8 (3165211)

10 embolism/ or exp pulmonary embolism/ or exp thromboembolism/ (103380)

11 exp Thrombosis/ (130421)

12 (DVT or VTE or PE).ti,ab. (63995)

13 ((venous or vein or pulmonary or lung) adj3 (emboli* or thromb*)).mp. [mp=title, abstract, original title, name of substance word, subject heading word, floating sub-heading word, keyword heading word, organism supplementary concept word, protocol supplementary concept word, rare disease supplementary concept word, unique identifier, synonyms] (128907)

14 (DVT or VTE or PE or PTE).ti,ab. (66040)

15 or/10-14 (295147)

Annotation: VTE block

16 9 and 15 (131905)

Annotation: post op comps AND VTE

17 Appendectomy/ or exp Bariatric Surgery/ or exp Cholecystectomy/ or exp Colectomy/ or exp Gastrectomy/ or Hepatectomy/ or Herniorrhaphy/ or pancreatectomy/ or Pancreaticoduodenectomy/ or pancreaticojejunostomy/ or Splenectomy/ (194233)

18 General Surgery/ or exp digestive system surgical procedures/ (407450)

19 exp Digestive System/su or Cholecystitis/su or Gallbladder/su or exp Gallbladder Diseases/su or Hernia, Abdominal/su or Hernia, Inguinal/su or exp Hernia, Ventral/su or exp Intestinal Diseases/su or exp Liver Diseases/su or exp Pancreas/su or exp Pancreatic Diseases/su or Spleen/su or exp Splenic Diseases/su or exp Stomach Diseases/su (334048)

20 (appendectom* or appendicectom* or colectomy* or proctocolectom* or cholecystectom* or duodenectom* or gastrectom* or hernioplast* or herniorrhaph* or herniotom* or jejunectom* or pancreatectom* or pancreaticojejunostom* or pancreaticoduodenectom* or duodenopancreatectom*).mp. [mp=title, abstract, original title, name of substance word, subject heading word, floating sub-heading word, keyword heading word, organism supplementary concept word, protocol supplementary concept word, rare disease supplementary concept word, unique identifier, synonyms] (162568)

21 ((surgery or resection* or excision* or repair* or operation* or laproscop* or laparoscop* or sleeve*) adj3 (abdominoperineal or perineal or anal* or anus or appendix or bowel* or colon* or duoden* or jejun* or ileal* or ileum* or jejuno?ileal or intestine* or gall bladder or gall?bladder or gastric or bariatric* or stomach or hernia or liver or adenoma or hepatoma* or hepatocellular* or rectal* or rectum)).mp. [mp=title, abstract, original title, name of substance word, subject heading word, floating sub-heading word, keyword heading word, organism supplementary concept word, protocol supplementary concept word, rare disease supplementary concept word, unique identifier, synonyms] (154351)

22 ((general or abdominal or major) adj3 (surgery or surgical)).mp. (100037)

23 (prolapse adj3 rectal).mp. [mp=title, abstract, original title, name of substance word, subject heading word, floating sub-heading word, keyword heading word, organism supplementary concept word, protocol supplementary concept word, rare disease supplementary concept word, unique identifier, synonyms] (3729)

24 (Rectopexy or rectosigmoidectom* or sigmoidectom* or DHoore or d'hoore or Delorme or Altemeier).mp. (2266)

25 or/17-24 (754910)

26 16 and 25 (9517)

27 9 and 15 and 25 (9517)

28 limit 27 to ed=20190301-20201027 (749)

29 limit 27 to yr="2019 -Current" (779)

30 28 or 29 (978)

→31 search for transplant* in title or keyword field in Endnote (150)

→32 30 NOT 31 in Endnote (828)

Database: Embase <1974 to 2020 October 26>

Search Strategy:

--------------------------------------------------------------------------------

1 ((chemoprophylax* or chemoprophylactic* or prophylax* or prophylactic*) and (venous or vein or thromb*)).ti,ab. (32192)

2 (prevent* adj3 (venous or vein or thromb*)).mp. (31383)

3 (thromboprophylax* or thromboprophylactic*).mp. (8518)

4 *postoperative complication/co, ep, et, pc [Complication, Epidemiology, Etiology, Prevention] (38244)

5 exp *venous thromboembolism/co, ep, et, pc [Complication, Epidemiology, Etiology, Prevention] (19508)

6 thrombosis prevention/ (11876)

7 postoperative complication/ep [Epidemiology] (10491)

8 *venous thromboembolism/ (16821)

9 *deep vein thrombosis/ (17253)

10 venous thromboembolism/ep [Epidemiology] (1520)

11 risk factor/ (1068091)

12 (ep or co).fs. and (venous or thromb* or bleed* or haemorr* or hemorr*).ti,ab. (234412)

13 (risk* or high-risk or incidence* or meta?analysis or analysis or complication* or outcome* or safety or versus or thrombosis or transfusion* or adverse or bleed* or haemorr* or hemorr*).ti. (3136902)

14 or/1-13 (4013485)

Annotation: post op VTE comp

15 exp thromboembolism/ (490180)

16 (DVT or VTE or PE or PTE).ti,ab. (97278)

17 ((venous or vein or pulmonary or lung) adj3 (emboli* or thromb*)).mp. (243933)

18 or/15-17 (565928)

Annotation: VTE broad

19 14 and 18 (252700)

Annotation: risk of post-op VTE

20 general surgery/ (16045)

21 exp abdominal surgery/ (795673)

22 exp gastrointestinal surgery/ (366003)

23 cholecystitis/su [Surgery] (3367)

24 gallbladder disease/su [Surgery] (1815)

25 exp abdominal wall hernia/su [Surgery] (16913)

26 exp enteropathy/su [Surgery] (135296)

27 exp enteropathy/su [Surgery] (135296)

28 exp liver disease/su [Surgery] (66737)

29 exp pancreas disease/su [Surgery] (37125)

30 exp spleen disease/su [Surgery] (7420)

31 exp stomach disease/su [Surgery] (46514)

32 ((general or abdominal or major) adj3 (surgery or surgical)).mp. (115366)

33 (prolapse adj3 rectal).mp. [mp=title, abstract, heading word, drug trade name, original title, device manufacturer, drug manufacturer, device trade name, keyword, floating subheading word, candidate term word] (3629)

34 (rectopexy or proctopexy or rectosigmoidectom* or sigmoidectom* or DHoore or d'hoore or Delorme or Altemeier).mp. (6344)

35 (surgery or resection* or excision* or repair* or operation* or laproscop* or sleeve*).mp. and (exp digestive system/ or exp spleen/) [mp=title, abstract, heading word, drug trade name, original title, device manufacturer, drug manufacturer, device trade name, keyword, floating subheading word, candidate term word] (258623)

36 (appendectom* or appendicectom* or colectomy* or proctocolectom* or cholecystectom* or duodenectom* or gastrectom* or hernioplast* or herniorrhaph* or herniotom* or jejunectom* or pancreatectom* or pancreaticojejunostom* or pancreaticoduodenectom* or duodenopancreatectom*).mp. (217703)

37 ((surgery or resection* or excision* or repair* or operation* or laproscop* or laparoscop* or sleeve*) adj3 (abdominoperineal or anal* or anus or appendix or bowel* or colon* or duoden* or jejun* or ileal* or ileum* or jejuno?ileal or intestine* or gall bladder or gall?bladder or gastric or bariatric* or stomach or hernia or liver or adenoma or hepatoma* or hepatocellular* or rectal* or rectum)).mp. (312571)

38 or/20-37 (1141588)

39 14 and 18 and 38 (26028)

40 exp animals/ or exp invertebrate/ or animal experiment/ or animal model/ or animal tissue/ or animal cell/ or nonhuman/ (28182878)

41 human/ or normal human/ or human cell/ (21665531)

42 40 and 41 (21599953)

43 40 not 42 (6582925)

44 39 not 43 (25755)

45 exp controlled clinical trial/ (817708)

46 44 not 45 (24679)

47 clinical study/ (156238)

48 case control study/ (163048)

49 family study/ (26140)

50 longitudinal study/ (146898)

51 retrospective study/ (984448)

52 prospective study/ (638840)

53 cohort analysis/ (631612)

54 (Cohort adj (study or studies)).mp. (322816)

55 (Case control adj (study or studies)).tw. (137939)

56 (follow up adj (study or studies)).tw. (64541)

57 (observational adj (study or studies)).tw. (175801)

58 (epidemiologic$ adj (study or studies)).tw. (108420)

59 (cross sectional adj (study or studies)).tw. (231148)

60 or/47-59 (2849250)

61 46 and 60 (6858)

62 (prognosis or prognostic or predict* or risk*).mp. [mp=title, abstract, heading word, drug trade name, original title, device manufacturer, drug manufacturer, device trade name, keyword, floating subheading word, candidate term word] (6352775)

63 (incidence* or outcome* or comparison* or complication*).ti. (1244456)

64 prevalence.mp. or prevalence/ (1092785)

65 baseline.mp. (980378)

66 or/62-65 (8208047)

67 46 and 66 (16930)

68 61 or 67 (18189)

69 transplant*.ti,kw,jw. (528976)

70 transplant*.ab. /freq=2 (353485)

71 69 or 70 (614781)

72 68 not 71 (14352)

73 limit 72 to em=201911-202052 (1333)

74 limit 72 to yr="2019 -Current" (2118)

75 73 or 74 (2274)

**Web of Science**

| # 19 | [**1,917**](http://apps.webofknowledge.com.libaccess.lib.mcmaster.ca/summary.do?product=WOS&doc=1&qid=20&SID=8A2W7pNdtCZ349dFcYN&search_mode=AdvancedSearch&update_back2search_link_param=yes) | #18  *Indexes=SCI-EXPANDED, SSCI, A&HCI, CPCI-S, CPCI-SSH, BKCI-S, BKCI-SSH, ESCI, CCR-EXPANDED, IC Timespan=2019-2020* |
| --- | --- | --- |
| # 18 | [**11,210**](http://apps.webofknowledge.com.libaccess.lib.mcmaster.ca/summary.do?product=WOS&doc=1&qid=19&SID=8A2W7pNdtCZ349dFcYN&search_mode=AdvancedSearch&update_back2search_link_param=yes) | #16 not #17  *Indexes=SCI-EXPANDED, SSCI, A&HCI, CPCI-S, CPCI-SSH, BKCI-S, BKCI-SSH, ESCI, CCR-EXPANDED, IC Timespan=All years* |
| # 17 | [**668,584**](http://apps.webofknowledge.com.libaccess.lib.mcmaster.ca/summary.do?product=WOS&doc=1&qid=18&SID=8A2W7pNdtCZ349dFcYN&search_mode=AdvancedSearch&update_back2search_link_param=yes) | TS=transplant*  *Indexes=SCI-EXPANDED, SSCI, A&HCI, CPCI-S, CPCI-SSH, BKCI-S, BKCI-SSH, ESCI, CCR-EXPANDED, IC Timespan=All years* |
| # 16 | [**12,310**](http://apps.webofknowledge.com.libaccess.lib.mcmaster.ca/summary.do?product=WOS&doc=1&qid=17&SID=8A2W7pNdtCZ349dFcYN&search_mode=AdvancedSearch&update_back2search_link_param=yes) | #15 AND #14  *Indexes=SCI-EXPANDED, SSCI, A&HCI, CPCI-S, CPCI-SSH, BKCI-S, BKCI-SSH, ESCI, CCR-EXPANDED, IC Timespan=All years* |
| # 15 | [**18,007,315**](http://apps.webofknowledge.com.libaccess.lib.mcmaster.ca/summary.do?product=WOS&doc=1&qid=16&SID=8A2W7pNdtCZ349dFcYN&search_mode=AdvancedSearch&update_back2search_link_param=yes) | TS=(cohort or observational or cross-sectional or longitudinal NEAR/2 study or studies)  *Indexes=SCI-EXPANDED, SSCI, A&HCI, CPCI-S, CPCI-SSH, BKCI-S, BKCI-SSH, ESCI, CCR-EXPANDED, IC Timespan=All years* |
| # 14 | [**34,017**](http://apps.webofknowledge.com.libaccess.lib.mcmaster.ca/summary.do?product=WOS&doc=1&qid=15&SID=8A2W7pNdtCZ349dFcYN&search_mode=AdvancedSearch&update_back2search_link_param=yes) | #13 AND #8  *Indexes=SCI-EXPANDED, SSCI, A&HCI, CPCI-S, CPCI-SSH, BKCI-S, BKCI-SSH, ESCI, CCR-EXPANDED, IC Timespan=All years* |
| # 13 | [**1,948,488**](http://apps.webofknowledge.com.libaccess.lib.mcmaster.ca/summary.do?product=WOS&doc=1&qid=14&SID=8A2W7pNdtCZ349dFcYN&search_mode=AdvancedSearch&update_back2search_link_param=yes) | #12 OR #11 OR #10 OR #9  *Indexes=SCI-EXPANDED, SSCI, A&HCI, CPCI-S, CPCI-SSH, BKCI-S, BKCI-SSH, ESCI, CCR-EXPANDED, IC Timespan=All years* |
| # 12 | [**1,417,082**](http://apps.webofknowledge.com.libaccess.lib.mcmaster.ca/summary.do?product=WOS&doc=1&qid=13&SID=8A2W7pNdtCZ349dFcYN&search_mode=AdvancedSearch&update_back2search_link_param=yes) | TI=(complication* or outcome* or safety or versus or thrombosis or transfusion* or adverse or bleed* or haemorr* or hemorr*)  *Indexes=SCI-EXPANDED, SSCI, A&HCI, CPCI-S, CPCI-SSH, BKCI-S, BKCI-SSH, ESCI, CCR-EXPANDED, IC Timespan=All years* |
| # 11 | [**6,802**](http://apps.webofknowledge.com.libaccess.lib.mcmaster.ca/summary.do?product=WOS&doc=1&qid=12&SID=8A2W7pNdtCZ349dFcYN&search_mode=AdvancedSearch&update_back2search_link_param=yes) | TS=(thromboprophylax* or thromboprophylactic*)  *Indexes=SCI-EXPANDED, SSCI, A&HCI, CPCI-S, CPCI-SSH, BKCI-S, BKCI-SSH, ESCI, CCR-EXPANDED, IC Timespan=All years* |
| # 10 | [**636,253**](http://apps.webofknowledge.com.libaccess.lib.mcmaster.ca/summary.do?product=WOS&doc=1&qid=11&SID=8A2W7pNdtCZ349dFcYN&search_mode=AdvancedSearch&update_back2search_link_param=yes) | TS=(prevent* NEAR/3 venous or vein or thromb*)  *Indexes=SCI-EXPANDED, SSCI, A&HCI, CPCI-S, CPCI-SSH, BKCI-S, BKCI-SSH, ESCI, CCR-EXPANDED, IC Timespan=All years* |
| # 9 | [**6,619**](http://apps.webofknowledge.com.libaccess.lib.mcmaster.ca/summary.do?product=WOS&doc=1&qid=10&SID=8A2W7pNdtCZ349dFcYN&search_mode=AdvancedSearch&update_back2search_link_param=yes) | TS=((chemoprophylax* or chemoprophylactic*or prophylax* or prophylactic*) and (venous or vein or thromb*) )  *Indexes=SCI-EXPANDED, SSCI, A&HCI, CPCI-S, CPCI-SSH, BKCI-S, BKCI-SSH, ESCI, CCR-EXPANDED, IC Timespan=All years* |
| # 8 | [**54,900**](http://apps.webofknowledge.com.libaccess.lib.mcmaster.ca/summary.do?product=WOS&doc=1&qid=9&SID=8A2W7pNdtCZ349dFcYN&search_mode=AdvancedSearch&update_back2search_link_param=yes) | #7 AND #4  *Indexes=SCI-EXPANDED, SSCI, A&HCI, CPCI-S, CPCI-SSH, BKCI-S, BKCI-SSH, ESCI, CCR-EXPANDED, IC Timespan=All years* |
| # 7 | [**548,889**](http://apps.webofknowledge.com.libaccess.lib.mcmaster.ca/summary.do?product=WOS&doc=1&qid=8&SID=8A2W7pNdtCZ349dFcYN&search_mode=AdvancedSearch&update_back2search_link_param=yes) | #6 OR #5  *Indexes=SCI-EXPANDED, SSCI, A&HCI, CPCI-S, CPCI-SSH, BKCI-S, BKCI-SSH, ESCI, CCR-EXPANDED, IC Timespan=All years* |
| # 6 | [**530,835**](http://apps.webofknowledge.com.libaccess.lib.mcmaster.ca/summary.do?product=WOS&doc=1&qid=7&SID=8A2W7pNdtCZ349dFcYN&search_mode=AdvancedSearch&update_back2search_link_param=yes) | TI=(venous or vein or pulmonary or lung NEAR/3 emboli* or thromb*)  *Indexes=SCI-EXPANDED, SSCI, A&HCI, CPCI-S, CPCI-SSH, BKCI-S, BKCI-SSH, ESCI, CCR-EXPANDED, IC Timespan=All years* |
| # 5 | [**33,498**](http://apps.webofknowledge.com.libaccess.lib.mcmaster.ca/summary.do?product=WOS&doc=1&qid=6&SID=8A2W7pNdtCZ349dFcYN&search_mode=AdvancedSearch&update_back2search_link_param=yes) | TS=(DVT or VTE or PE or PTE)  *Indexes=SCI-EXPANDED, SSCI, A&HCI, CPCI-S, CPCI-SSH, BKCI-S, BKCI-SSH, ESCI, CCR-EXPANDED, IC Timespan=All years* |
| # 4 | [**5,084,933**](http://apps.webofknowledge.com.libaccess.lib.mcmaster.ca/summary.do?product=WOS&doc=1&qid=5&SID=8A2W7pNdtCZ349dFcYN&search_mode=AdvancedSearch&update_back2search_link_param=yes) | #3 OR #2 OR #1  *Indexes=SCI-EXPANDED, SSCI, A&HCI, CPCI-S, CPCI-SSH, BKCI-S, BKCI-SSH, ESCI, CCR-EXPANDED, IC Timespan=All years* |
| # 3 | [**44,375**](http://apps.webofknowledge.com.libaccess.lib.mcmaster.ca/summary.do?product=WOS&doc=1&qid=4&SID=8A2W7pNdtCZ349dFcYN&search_mode=AdvancedSearch&update_back2search_link_param=yes) | TI=(appendectom* or appendicectom* or colectomy* or proctocolectom* or cholecystectom* or duodenectom* or gastrectom* or hernioplast* or herniorrhaph* or herniotom* or jejunectom* or pancreatecom* or pancreaticojejunostom* or pancreaticoduodenectom* or duodenopancreatectom* or rectopexy or rectosigmoidectom* or sigmoidectom* or DHoore or d'hoore or Delorme or Altemeier)  *Indexes=SCI-EXPANDED, SSCI, A&HCI, CPCI-S, CPCI-SSH, BKCI-S, BKCI-SSH, ESCI, CCR-EXPANDED, IC Timespan=All years* |
| # 2 | [**4,670,456**](http://apps.webofknowledge.com.libaccess.lib.mcmaster.ca/summary.do?product=WOS&doc=1&qid=3&SID=8A2W7pNdtCZ349dFcYN&search_mode=AdvancedSearch&update_back2search_link_param=yes) | TI=(surgery or resection* or excision* or repair* or operation* or prolapse* or laproscop* or laparoscop* or sleeve* NEAR/3 abdominoperineal or anal* or anus or appendix or bowel* or colon* or duoden* or jejun* ileal* or ileum* or jejuno?ileal or intestine* or gall bladder or gall?bladder or gastric or bariatric* or stomach or hernia or liver or adenoma or hepatoma* or hepatocellular* or rectal* or rectum)  *Indexes=SCI-EXPANDED, SSCI, A&HCI, CPCI-S, CPCI-SSH, BKCI-S, BKCI-SSH, ESCI, CCR-EXPANDED, IC Timespan=All years* |
| # 1 | [**485,031**](http://apps.webofknowledge.com.libaccess.lib.mcmaster.ca/summary.do?product=WOS&doc=1&qid=2&SID=8A2W7pNdtCZ349dFcYN&search_mode=AdvancedSearch&update_back2search_link_param=yes) | TI= (general or abdominal or major NEAR/3 surgery or surgical)  *Indexes=SCI-EXPANDED, SSCI, A&HCI, CPCI-S, CPCI-SSH, BKCI-S, BKCI-SSH, ESCI, CCR-EXPANDED, IC Timespan=All years* |
|  |  |  |

**Google Scholar**

Google Search update Aug 5, 2021

We queried Google Scholar using Harzig’s PublishorPerish for macOSVersion: 7.33.3373 (28 May 2021)

https://harzing.com/resources/publish-or-perish/os-x

risk AND (embolism or thrombosis or DVT or VTE or PE or PTE) and "general surgery"

and years 2019-2020

yield =26

**Supplementary Methods**

**2. Search history for baseline risk of VTE and major bleeding in gynecological surgery**

**MEDLINE**

Database: OVID Medline Epub Ahead of Print, In-Process & Other Non-Indexed Citations, Ovid MEDLINE(R) Daily and Ovid MEDLINE(R) 1946 to October 22, 2019

Search Strategy:

--------------------------------------------------------------------------------

1 exp Gynecologic Surgical Procedures/ or exp Genital Diseases, Female/su or exp Genitalia, Female/su or Uterine Artery/su or Gynecology/su or Female Urogenital Diseases/su or Oocyte Retrieval/ or exp Ovarian Cysts/su or exp Pregnancy, Ectopic/su or "Dilatation and Curettage"/ or exp Pregnancy Complications/su or Hysteroscopy/ or exp Pelvic Organ Prolapse/su (145163)

2 Urethra/su or Urinary Bladder/su or exp Urinary Incontinence/su or Suburethral Slings/ or Sterilization reversal/ (24032)

3 limit 2 to female (13596)

4 1 or 3 (154601)

Annotation: MeSH for gyne surgical procedures

5 ((gyn?ecolog* or ovar* or vulv* or vagin* or transvagin* or adnex* or fibroid* or myoma or leiomyoma or pelvic or perineal or endometri* or cervic* or cervix or uterus or uterin* or fallop* or curettage or labia* or clitoris) adj3 (resect* or excision* or ablation or conisation or conization or endoscop* or embol* or operation or torsion or amputation or rupture or cytoreduc*or repair or fixation or reconstruct* or surgery or surgeries or procedure* or removal)).mp. (69126)

6 (cervicopex* or cervicectom* or colpectom* or colporrhaph or colpopex* or colposcop* or colposuspen* or colpotom* or culdoplast* or exenteration* or fibroidectom* or fimbriectom* or hysterectom* or hysteropex* or hysteroscop* or hysterosalpingo* or LAVH or LEEP or LLETZ or USLS or myomectom* or oophorectom* or omentectom* or ovariectom* or perineoplast* or perineorrhaph* or sacrocolpop* or sacropex* or salpingectom* or salpingostom* or salpingo-oophorectom* or trachelectom* or tubectom* or tuboplast* or uteroscop* or vaginotom* or vaginoplast* or vulvectom* or Wertheim or labiaplast*).mp. [mp=title, abstract, original title, name of substance word, subject heading word, floating sub-heading word, keyword heading word, organism supplementary concept word, protocol supplementary concept word, rare disease supplementary concept word, unique identifier, synonyms] (117670)

7 (oocyte* adj2 (retriev* or aspirat* or collect*)).mp. (8122)

8 (Tubal adj2 (excision or ligation or occlusion or ring or rings or sterilization or reanastomosis)).mp. (6395)

9 ((vaginal or pubovaginal or transvaginal or retropubic or transobturator or prolapse) adj3 (tape* or sling* or mesh*)).mp. (4077)

10 (TVT or mini-sling or miniarc or bulkamid).mp. [mp=title, abstract, original title, name of substance word, subject heading word, floating sub-heading word, keyword heading word, organism supplementary concept word, protocol supplementary concept word, rare disease supplementary concept word, unique identifier, synonyms] (1768)

11 or/4-10 (237127)

Annotation: gyne surgical procedures

12 (Gyneco* adj3 surg*).jw. (13810)

13 (excision adj3 loop).mp. (1273)

14 ((ovar* or luteum or luteal) adj3 (cystectom* or resect* or enucleat* or excision* or ablation or conisation or conization or endoscop* or embol* or operation or torsion or amputation or rupture or cytoreduc*or repair or fixation or reconstruct* or surgery or surgeries or procedure* or removal)).mp. [mp=title, abstract, original title, name of substance word, subject heading word, floating sub-heading word, keyword heading word, organism supplementary concept word, protocol supplementary concept word, rare disease supplementary concept word, unique identifier, synonyms] (7838)

15 ((ectopic or extrauterin* or tubal*) adj3 pregnan* adj3 (cystectom* or resect* or enucleat* or excision* or ablation or conisation or conization or endoscop* or embol* or operation or torsion or amputation or rupture or cytoreduc*or repair or fixation or reconstruct* or surgery or surgeries or procedure* or removal)).mp. [mp=title, abstract, original title, name of substance word, subject heading word, floating sub-heading word, keyword heading word, organism supplementary concept word, protocol supplementary concept word, rare disease supplementary concept word, unique identifier, synonyms] (897)

16 ((sacrospinous or uterosacral or sacral ligament) adj3 (fixat* or suspens* or plicat*)).mp. (562)

17 ((dilatation or curettage or evacuation) adj5 (conception or cervix or cervic* or uter* or pregnancy or placent*)).mp. (5458)

18 or/4-17 (253976)

Annotation: Gyne surgery

19 ((chemoprophylax* chemoprophylactic*or prophylax* or prophylactic*) and (venous or vein or thromb*)).ti,ab. (6549)

20 (prevent* adj3 (venous or vein or thromb*)).mp. (14882)

21 (thromboprophylax* or thromboprophylactic*).mp. [mp=title, abstract, original title, name of substance word, subject heading word, floating sub-heading word, keyword heading word, organism supplementary concept word, protocol supplementary concept word, rare disease supplementary concept word, unique identifier, synonyms] (4679)

22 *Postoperative Complications/ (153012)

23 Postoperative Complications/ep, et, pc [Epidemiology, Etiology, Prevention & Control] (136920)

24 Risk Factors/ (788290)

25 (ep or ae).fs. and (venous or thromb* or bleed* or haemorr* or hemorr*).ti,ab. (198285)

26 (risk* or high-risk or incidence* or meta?analysis or analysis or complication* or outcome* or safety or versus or thrombosis or transfusion* or adverse or bleed* or haemorr* or hemorr*).ti. (2221404)

27 or/19-26 (2953549)

28 embolism/ or exp pulmonary embolism/ or exp thromboembolism/ (99725)

29 exp Thrombosis/ (126529)

30 (DVT or VTE or PE).ti,ab. (57514)

31 ((venous or vein or pulmonary or lung) adj3 (emboli* or thromb*)).mp. [mp=title, abstract, original title, name of substance word, subject heading word, floating sub-heading word, keyword heading word, organism supplementary concept word, protocol supplementary concept word, rare disease supplementary concept word, unique identifier, synonyms] (121436)

32 (DVT or VTE or PE or PTE).ti,ab. (59347)

33 or/28-32 (279732)

Annotation: VTE block

34 27 and 33 (123296)

Annotation: post op comps AND VTE

35 18 and 34 (1969)

36 animals/ not humans/ (4602086)

37 35 not 36 (1939)

**EMBASE**

Database: Embase <1974 to 2019 October 22>

Search Strategy:

--------------------------------------------------------------------------------

1 exp gynecologic surgery/ or exp female genital system/su or exp gynecologic
disease/su or uterine artery/su or oocyte retrieval/ or exp ovary cyst/su or ectopic
pregnancy/su or "dilatation and curettage"/ or exp pregnancy complication/su or
hysteroscopy/ or exp pelvic organ prolapse/su or female sterilization reversal/
(206205)

2 exp urethra/su or bladder/su or exp bladder surgery/ or exp urethra surgery/ or
exp urine incontinence/su or exp suburethral sling/ or sterilization reversal/
(60598)

3 limit 2 to female (28581)

4 1 or 3 (227889)

Annotation: gyne surgery Emtree terms

5 ((gyn?ecolog* or ovar* or vulv* or vagin* or transvagin* or adnex* or fibroid*
or myoma or leiomyoma or pelvic or perineal or endometri* or cervic* or cervix or
uterus or uterin* or fallop* or curettage or labia* or clitoris) adj3 (resect* or
excision* or ablation or conisation or conization or endoscop* or embol* or operation
or torsion or amputation or rupture or cytoreduc*or repair or fixation or
reconstruct* or surgery or surgeries or procedure* or removal)).mp. (107838)

6 (cervicopex* or cervicectom* or colpectom* or colporrhaph or colpopex* or
colposcop* or colposuspen* or colpotom* or culdoplast* or exenteration* or
fibroidectom* or fimbriectom* or hysterectom* or hysteropex* or hysteroscop* or
hysterosalpingo* or LAVH or LEEP or LLETZ or USLS or myomectom* or oophorectom* or
omentectom* or ovariectom* or perineoplast* or perineorrhaph* or sacrocolpop* or
sacropex* or salpingectom* or salpingostom* or salpingo-oophorectom* or trachelectom*
or tubectom* or tuboplast* or uteroscop* or vaginotom* or vaginoplast* or vulvectom*
or Wertheim or labiaplast*).mp. [mp=title, abstract, heading word, drug trade name,
original title, device manufacturer, drug manufacturer, device trade name, keyword,
floating subheading word, candidate term word] (176353)

7 (oocyte* adj2 (retriev* or aspirat* or collect*)).mp. (15429)

8 (Tubal adj2 (excision or ligation or occlusion or ring or rings or
sterilization or reanastomosis)).mp. (5310)

9 ((vaginal or pubovaginal or transvaginal or retropubic or transobturator or
prolapse) adj3 (tape* or sling* or mesh*)).mp. (9719)

10 (TVT or mini-sling or miniarc or bulkamid).mp. [mp=title, abstract, heading
word, drug trade name, original title, device manufacturer, drug manufacturer, device
trade name, keyword, floating subheading word, candidate term word] (3789)

11 (Gyneco* adj3 surg*).jw. (11925)

12 (excision adj3 loop).mp. (1932)

13 ((ovar* or luteum or luteal) adj3 (cystectom* or resect* or enucleat* or
excision* or ablation or conisation or conization or endoscop* or embol* or operation
or torsion or amputation or rupture or cytoreduc*or repair or fixation or
reconstruct* or surgery or surgeries or procedure* or removal)).mp. [mp=title,
abstract, heading word, drug trade name, original title, device manufacturer, drug
manufacturer, device trade name, keyword, floating subheading word, candidate term
word] (12343)

14 ((ectopic or extrauterin* or tubal*) adj3 pregnan* adj3 (cystectom* or resect*
or enucleat* or excision* or ablation or conisation or conization or endoscop* or
embol* or operation or torsion or amputation or rupture or cytoreduc*or repair or
fixation or reconstruct* or surgery or surgeries or procedure* or removal)).mp.
[mp=title, abstract, heading word, drug trade name, original title, device
manufacturer, drug manufacturer, device trade name, keyword, floating subheading
word, candidate term word] (1145)

15 ((sacrospinous or uterosacral or sacral ligament) adj3 (fixat* or suspens* or
plicat*)).mp. (1459)

16 ((dilatation or curettage or evacuation) adj5 (conception or cervix or cervic*
or uter* or pregnancy or placent*)).mp. (9244)

17 or/4-16 (342336)

18 ((chemoprophylax* or chemoprophylactic* or prophylax* or prophylactic*) and
(venous or vein or thromb*)).ti,ab. (30274)

19 (prevent* adj3 (venous or vein or thromb*)).mp. (29584)

20 (thromboprophylax* or thromboprophylactic*).mp. (7851)

21 *postoperative complication/co, ep, et, pc [Complication, Epidemiology,
Etiology, Prevention] (35783)

22 exp *venous thromboembolism/co, ep, et, pc [Complication, Epidemiology,
Etiology, Prevention] (18819)

23 thrombosis prevention/ (10994)

24 postoperative complication/ep [Epidemiology] (9654)

25 *venous thromboembolism/ (15514)

26 *deep vein thrombosis/ (16468)

27 venous thromboembolism/ep [Epidemiology] (1420)

28 risk factor/ (986601)

29 (ep or co).fs. and (venous or thromb* or bleed* or haemorr* or hemorr*).ti,ab.
(227234)

30 (risk* or high-risk or incidence* or meta?analysis or analysis or
complication* or outcome* or safety or versus or thrombosis or transfusion* or
adverse or bleed* or haemorr* or hemorr*).ti. (2894464)

31 or/18-30 (3715670)

Annotation: post op VTE comp

32 exp thromboembolism/ (464121)

33 (DVT or VTE or PE or PTE).ti,ab. (89132)

34 ((venous or vein or pulmonary or lung) adj3 (emboli* or thromb*)).mp. (228335)

35 or/32-34 (533811)

Annotation: VTE broad

36 31 and 35 (237706)

Annotation: risk of post-op VTE

37 17 and 36 (4881)

Annotation: Gyne surgery and risk of VTE

38 exp animals/ or exp invertebrate/ or animal experiment/ or animal model/ or
animal tissue/ or animal cell/ or nonhuman/ (26654410)

39 human/ or normal human/ or human cell/ (20374078)

40 38 and 39 (20313407)

41 38 not 40 (6341003)

42 37 not 41 (4840)

43 exp controlled clinical trial/ (764645)

44 42 not 43 (4609)

45 clinical study/ (154856)

46 case control study/ (147809)

47 family study/ (26156)

48 longitudinal study/ (132286)

49 retrospective study/ (842160)

50 prospective study/ (560957)

51 cohort analysis/ (521571)

52 (Cohort adj (study or studies)).mp. (281753)

53 (Case control adj (study or studies)).tw. (128099)

54 (follow up adj (study or studies)).tw. (61467)

55 (observational adj (study or studies)).tw. (154443)

56 (epidemiologic$ adj (study or studies)).tw. (103091)

57 (cross sectional adj (study or studies)).tw. (200462)

58 or/45-57 (2525427)

59 44 and 58 (1376)

60 (prognosis or prognostic or predict* or risk*).mp. [mp=title, abstract,
heading word, drug trade name, original title, device manufacturer, drug
manufacturer, device trade name, keyword, floating subheading word, candidate term
word] (5862135)

61 (incidence* or outcome* or comparison* or complication*).ti. (1154717)

62 prevalence.mp. or prevalence/ (1007022)

63 baseline.mp. (899249)

64 or/60-63 (7590919)

65 44 and 64 (3267)

66 59 or 65 (3483)

**Web of Science**

| **Set** | **Results** | **Save History / Create AlertOpen Saved History** |
| --- | --- | --- |
| # 26 | [**4,151**](http://apps.webofknowledge.com/summary.do?product=WOS&doc=1&qid=67&SID=5BBCfqNCf2rZ3xmsox3&search_mode=CombineSearches&update_back2search_link_param=yes) | #25 AND #9  *Indexes=SCI-EXPANDED, SSCI, A&HCI, CPCI-S, CPCI-SSH, ESCI Timespan=1976-2019* |
| # 25 | [**149,113**](http://apps.webofknowledge.com/summary.do?product=WOS&doc=1&qid=66&SID=5BBCfqNCf2rZ3xmsox3&search_mode=CombineSearches&update_back2search_link_param=yes) | #24 OR #23 OR #22 OR #21 OR #20 OR #19 OR #18 OR #17 OR #16 OR #15 OR #14 OR #13 OR #12 OR #11 OR #10  *Indexes=SCI-EXPANDED, SSCI, A&HCI, CPCI-S, CPCI-SSH, ESCI Timespan=1976-2019* |
| # 24 | [**3,475**](http://apps.webofknowledge.com/summary.do?product=WOS&doc=1&qid=65&SID=5BBCfqNCf2rZ3xmsox3&search_mode=AdvancedSearch&update_back2search_link_param=yes) | TS=((dilatation or curettage or evacuation) near/5 (conception or cervix or cervic* or uter* or pregnancy or placent*))  *Indexes=SCI-EXPANDED, SSCI, A&HCI, CPCI-S, CPCI-SSH, ESCI Timespan=1976-2019* |
| # 23 | [**1,004**](http://apps.webofknowledge.com/summary.do?product=WOS&doc=1&qid=61&SID=5BBCfqNCf2rZ3xmsox3&search_mode=AdvancedSearch&update_back2search_link_param=yes) | TS=((sacrospinous or uterosacral or sacral) NEAR/3 (fixat* or suspens* or plicat*))  *Indexes=SCI-EXPANDED, SSCI, A&HCI, CPCI-S, CPCI-SSH, ESCI Timespan=1976-2019* |
| # 22 | [**469**](http://apps.webofknowledge.com/summary.do?product=WOS&doc=1&qid=58&SID=5BBCfqNCf2rZ3xmsox3&search_mode=AdvancedSearch&update_back2search_link_param=yes) | TS=(((ectopic or extrauterin* or tubal*) NEAR/3 (pregnan*) NEAR/3 (resect* or excision* or ablation or repair or fixation or reconstruct* or surger* or procedure* or removal)))  *Indexes=SCI-EXPANDED, SSCI, A&HCI, CPCI-S, CPCI-SSH, ESCI Timespan=1976-2019* |
| # 21 | [**106**](http://apps.webofknowledge.com/summary.do?product=WOS&doc=1&qid=56&SID=5BBCfqNCf2rZ3xmsox3&search_mode=AdvancedSearch&update_back2search_link_param=yes) | TS=((luteum or luteal) NEAR/3 (resect* or excision* or ablation or coni?ation or endoscop* or embol* or repair or fixation or reconstruct* or surger* or procedure* or removal))  *Indexes=SCI-EXPANDED, SSCI, A&HCI, CPCI-S, CPCI-SSH, ESCI Timespan=1976-2019* |
| # 20 | [**1,826**](http://apps.webofknowledge.com/summary.do?product=WOS&doc=1&qid=55&SID=5BBCfqNCf2rZ3xmsox3&search_mode=AdvancedSearch&update_back2search_link_param=yes) | TS=((fallop* or curettage or labia* or clitoris) NEAR/3 (resect* or excision* or ablation or coni?ation or endoscop* or embol* or repair or fixation or reconstruct* or surger* or procedure* or removal))  *Indexes=SCI-EXPANDED, SSCI, A&HCI, CPCI-S, CPCI-SSH, ESCI Timespan=1976-2019* |
| # 19 | [**21,083**](http://apps.webofknowledge.com/summary.do?product=WOS&doc=1&qid=54&SID=5BBCfqNCf2rZ3xmsox3&search_mode=AdvancedSearch&update_back2search_link_param=yes) | TS=((endometri* or cervic* or cervix or uterus or uterin*) NEAR/3 (resect* or excision* or ablation or coni?ation or endoscop* or embol* or repair or fixation or reconstruct* or surger* or procedure* or removal))  *Indexes=SCI-EXPANDED, SSCI, A&HCI, CPCI-S, CPCI-SSH, ESCI Timespan=1976-2019* |
| # 18 | [**14,311**](http://apps.webofknowledge.com/summary.do?product=WOS&doc=1&qid=53&SID=5BBCfqNCf2rZ3xmsox3&search_mode=AdvancedSearch&update_back2search_link_param=yes) | TS=((fibroid* or myoma or leiomyoma or pelvic or perineal) NEAR/3 (resect* or excision* or ablation or coni?ation or endoscop* or embol* or repair or fixation or reconstruct* or surger* or procedure* or removal))  *Indexes=SCI-EXPANDED, SSCI, A&HCI, CPCI-S, CPCI-SSH, ESCI Timespan=1976-2019* |
| # 17 | [**14,718**](http://apps.webofknowledge.com/summary.do?product=WOS&doc=1&qid=52&SID=5BBCfqNCf2rZ3xmsox3&search_mode=AdvancedSearch&update_back2search_link_param=yes) | TS=((ovar* or vulv* or vagin* or transvagin* or adnex*) NEAR/3 (resect* or excision* or ablation or coni?ation or endoscop* or embol* or repair or fixation or reconstruct* or surger* or procedure* or removal))  *Indexes=SCI-EXPANDED, SSCI, A&HCI, CPCI-S, CPCI-SSH, ESCI Timespan=1976-2019* |
| # 16 | [**1,524**](http://apps.webofknowledge.com/summary.do?product=WOS&doc=1&qid=41&SID=5BBCfqNCf2rZ3xmsox3&search_mode=AdvancedSearch&update_back2search_link_param=yes) | TS=(excision near/3 loop)  *Indexes=SCI-EXPANDED, SSCI, A&HCI, CPCI-S, CPCI-SSH, ESCI Timespan=1976-2019* |
| # 15 | [**2,597**](http://apps.webofknowledge.com/summary.do?product=WOS&doc=1&qid=39&SID=5BBCfqNCf2rZ3xmsox3&search_mode=AdvancedSearch&update_back2search_link_param=yes) | TS=(TVT or mini-sling or miniarc or bulkamid)  *Indexes=SCI-EXPANDED, SSCI, A&HCI, CPCI-S, CPCI-SSH, ESCI Timespan=1976-2019* |
| # 14 | [**5,985**](http://apps.webofknowledge.com/summary.do?product=WOS&doc=1&qid=38&SID=5BBCfqNCf2rZ3xmsox3&search_mode=AdvancedSearch&update_back2search_link_param=yes) | TS=((vaginal or pubovaginal or transvaginal or retropubic or transobturator or prolapse) near/3 (tape* or sling* or mesh*))  *Indexes=SCI-EXPANDED, SSCI, A&HCI, CPCI-S, CPCI-SSH, ESCI Timespan=1976-2019* |
| # 13 | [**3,214**](http://apps.webofknowledge.com/summary.do?product=WOS&doc=1&qid=37&SID=5BBCfqNCf2rZ3xmsox3&search_mode=AdvancedSearch&update_back2search_link_param=yes) | TS=(Tubal near/2 (excision or ligation or occlusion or ring or rings or sterilization or reanastomosis))  *Indexes=SCI-EXPANDED, SSCI, A&HCI, CPCI-S, CPCI-SSH, ESCI Timespan=1976-2019* |
| # 12 | [**7,781**](http://apps.webofknowledge.com/summary.do?product=WOS&doc=1&qid=36&SID=5BBCfqNCf2rZ3xmsox3&search_mode=AdvancedSearch&update_back2search_link_param=yes) | TS=(oocyte* near/2 (retriev* or aspirat* or collect*))  *Indexes=SCI-EXPANDED, SSCI, A&HCI, CPCI-S, CPCI-SSH, ESCI Timespan=1976-2019* |
| # 11 | [**92,640**](http://apps.webofknowledge.com/summary.do?product=WOS&doc=1&qid=34&SID=5BBCfqNCf2rZ3xmsox3&search_mode=AdvancedSearch&update_back2search_link_param=yes) | TS=(cervicopex* or cervicectom* or colpectom* or colporrhaph or colpopex* or colposcop* or colposuspen* or colpotom* or culdoplast* or exenteration* or fibroidectom* or fimbriectom* or hysterectom* or hysteropex* or hysteroscop* or hysterosalpingo* or LAVH or LEEP or LLETZ or USLS or myomectom* or oophorectom* or omentectom* or ovariectom* or perineoplast* or perineorrhaph* or sacrocolpop* or sacropex* or salpingectom* or salpingostom* or salpingo-oophorectom* or trachelectom* or tubectom* or tuboplast* or uteroscop* or vaginotom* or vaginoplast* or vulvectom* or Wertheim or labiaplast*)  *Indexes=SCI-EXPANDED, SSCI, A&HCI, CPCI-S, CPCI-SSH, ESCI Timespan=1976-2019* |
| # 10 | [**8,022**](http://apps.webofknowledge.com/summary.do?product=WOS&doc=1&qid=28&SID=5BBCfqNCf2rZ3xmsox3&search_mode=AdvancedSearch&update_back2search_link_param=yes) | TS=(((gyn$ecolog* or gynecol* or gynaecol*) NEAR/3 (surgery or surgical or surgeries)))  *Indexes=SCI-EXPANDED, SSCI, A&HCI, CPCI-S, CPCI-SSH, ESCI Timespan=1976-2019* |
| # 9 | [**734,781**](http://apps.webofknowledge.com/summary.do?product=WOS&doc=1&qid=18&SID=5BBCfqNCf2rZ3xmsox3&search_mode=CombineSearches&update_back2search_link_param=yes) | #8 AND #3  *Indexes=SCI-EXPANDED, SSCI, A&HCI, CPCI-S, CPCI-SSH, ESCI Timespan=1976-2019* |
| # 8 | [**4,773,217**](http://apps.webofknowledge.com/summary.do?product=WOS&doc=1&qid=13&SID=5BBCfqNCf2rZ3xmsox3&search_mode=CombineSearches&update_back2search_link_param=yes) | #7 OR #6 OR #5 OR #4  *Indexes=SCI-EXPANDED, SSCI, A&HCI, CPCI-S, CPCI-SSH, ESCI Timespan=1976-2019* |
| # 7 | [**4,451,610**](http://apps.webofknowledge.com/summary.do?product=WOS&doc=1&qid=12&SID=5BBCfqNCf2rZ3xmsox3&search_mode=AdvancedSearch&update_back2search_link_param=yes) | TS=(complication* or outcome* or safety or versus or thrombosis or transfusion* or adverse or bleed* or haemorr* or hemorr*)  *Indexes=SCI-EXPANDED, SSCI, A&HCI, CPCI-S, CPCI-SSH, ESCI Timespan=1976-2019* |
| # 6 | [**6,132**](http://apps.webofknowledge.com/summary.do?product=WOS&doc=1&qid=11&SID=5BBCfqNCf2rZ3xmsox3&search_mode=AdvancedSearch&update_back2search_link_param=yes) | TS=(thromboprophylax* or thromboprophylactic*)  *Indexes=SCI-EXPANDED, SSCI, A&HCI, CPCI-S, CPCI-SSH, ESCI Timespan=1976-2019* |
| # 5 | [**598,566**](http://apps.webofknowledge.com/summary.do?product=WOS&doc=1&qid=10&SID=5BBCfqNCf2rZ3xmsox3&search_mode=AdvancedSearch&update_back2search_link_param=yes) | TS=(prevent* NEAR/3 venous or vein or thromb*)  *Indexes=SCI-EXPANDED, SSCI, A&HCI, CPCI-S, CPCI-SSH, ESCI Timespan=1976-2019* |
| # 4 | [**6,115**](http://apps.webofknowledge.com/summary.do?product=WOS&doc=1&qid=9&SID=5BBCfqNCf2rZ3xmsox3&search_mode=AdvancedSearch&update_back2search_link_param=yes) | TS=((chemoprophylax* or chemoprophylactic*or prophylax* or prophylactic*) and (venous or vein or thromb*))  *Indexes=SCI-EXPANDED, SSCI, A&HCI, CPCI-S, CPCI-SSH, ESCI Timespan=1976-2019* |
| # 3 | [**1,143,587**](http://apps.webofknowledge.com/summary.do?product=WOS&doc=1&qid=7&SID=5BBCfqNCf2rZ3xmsox3&search_mode=CombineSearches&update_back2search_link_param=yes) | #2 OR #1  *Indexes=SCI-EXPANDED, SSCI, A&HCI, CPCI-S, CPCI-SSH, ESCI Timespan=1976-2019* |
| # 2 | [**1,130,387**](http://apps.webofknowledge.com/summary.do?product=WOS&doc=1&qid=6&SID=5BBCfqNCf2rZ3xmsox3&search_mode=AdvancedSearch&update_back2search_link_param=yes) | TS=(venous or vein or pulmonary or lung NEAR/3 emboli* or thromb*)  *Indexes=SCI-EXPANDED, SSCI, A&HCI, CPCI-S, CPCI-SSH, ESCI Timespan=1976-2019* |
| # 1 | [**30,145**](http://apps.webofknowledge.com/summary.do?product=WOS&doc=1&qid=5&SID=5BBCfqNCf2rZ3xmsox3&search_mode=AdvancedSearch&update_back2search_link_param=yes) | TS=(DVT or VTE or PE or PTE)  *Indexes=SCI-EXPANDED, SSCI, A&HCI, CPCI-S, CPCI-SSH, ESCI Timespan=1976-2019* |

**Google Scholar**

Query Google scholar using Publish or Perish 7.14.2619.7235

Windows (x64) edition, running on Windows 6.1.7601 (x64)

Harzing, A.W. (2007) Publish or Perish, available from <https://harzing.com/resources/publish-or-perish>

Search terms

Keywords: thromboembolism AND incidence AND (surgery OR resection OR excision OR operation) AND (gynecology or gynaecology)

Years: all

Data retrieval

Data source: Google Scholar

Search date: 2019-10-24 13:52:50 -0400

Cache date: 2019-10-24 14:14:59 -0400

Search result: [1027] Server error. (Selected 250 most highly ranked results)

## Search history update searches for baseline risk of VTE and major bleeding in gynecolological surgery

**MEDLINE**

Database: OVID Medline Epub Ahead of Print, In-Process & Other Non-Indexed Citations, Ovid MEDLINE(R) Daily and Ovid MEDLINE(R) 1946 to November 26, 2020

Search Strategy:

--------------------------------------------------------------------------------

1 exp Gynecologic Surgical Procedures/ or exp Genital Diseases, Female/su or exp Genitalia, Female/su or Uterine Artery/su or Gynecology/su or Female Urogenital Diseases/su or Oocyte Retrieval/ or exp Ovarian Cysts/su or exp Pregnancy, Ectopic/su or "Dilatation and Curettage"/ or exp Pregnancy Complications/su or Hysteroscopy/ or exp Pelvic Organ Prolapse/su (149776)

2 Urethra/su or Urinary Bladder/su or exp Urinary Incontinence/su or Suburethral Slings/ or Sterilization reversal/ (24685)

3 limit 2 to female (13998)

4 1 or 3 (159492)

Annotation: MeSH for gyne surgical procedures

5 ((gyn?ecolog* or ovar* or vulv* or vagin* or transvagin* or adnex* or fibroid* or myoma or leiomyoma or pelvic or perineal or endometri* or cervic* or cervix or uterus or uterin* or fallop* or curettage or labia* or clitoris) adj3 (resect* or excision* or ablation or conisation or conization or endoscop* or embol* or operation or torsion or amputation or rupture or cytoreduc*or repair or fixation or reconstruct* or surgery or surgeries or procedure* or removal)).mp. (73626)

6 (cervicopex* or cervicectom* or colpectom* or colporrhaph or colpopex* or colposcop* or colposuspen* or colpotom* or culdoplast* or exenteration* or fibroidectom* or fimbriectom* or hysterectom* or hysteropex* or hysteroscop* or hysterosalpingo* or LAVH or LEEP or LLETZ or USLS or myomectom* or oophorectom* or omentectom* or ovariectom* or perineoplast* or perineorrhaph* or sacrocolpop* or sacropex* or salpingectom* or salpingostom* or salpingo-oophorectom* or trachelectom* or tubectom* or tuboplast* or uteroscop* or vaginotom* or vaginoplast* or vulvectom* or Wertheim or labiaplast*).mp. [mp=title, abstract, original title, name of substance word, subject heading word, floating sub-heading word, keyword heading word, organism supplementary concept word, protocol supplementary concept word, rare disease supplementary concept word, unique identifier, synonyms] (122836)

7 (oocyte* adj2 (retriev* or aspirat* or collect*)).mp. (8720)

8 (Tubal adj2 (excision or ligation or occlusion or ring or rings or sterilization or reanastomosis)).mp. (6498)

9 ((vaginal or pubovaginal or transvaginal or retropubic or transobturator or prolapse) adj3 (tape* or sling* or mesh*)).mp. (4322)

10 (TVT or mini-sling or miniarc or bulkamid).mp. [mp=title, abstract, original title, name of substance word, subject heading word, floating sub-heading word, keyword heading word, organism supplementary concept word, protocol supplementary concept word, rare disease supplementary concept word, unique identifier, synonyms] (1852)

11 or/4-10 (247770)

Annotation: gyne surgical procedures

12 (Gyneco* adj3 surg*).jw. (13819)

13 (excision adj3 loop).mp. (1344)

14 ((ovar* or luteum or luteal) adj3 (cystectom* or resect* or enucleat* or excision* or ablation or conisation or conization or endoscop* or embol* or operation or torsion or amputation or rupture or cytoreduc*or repair or fixation or reconstruct* or surgery or surgeries or procedure* or removal)).mp. [mp=title, abstract, original title, name of substance word, subject heading word, floating sub-heading word, keyword heading word, organism supplementary concept word, protocol supplementary concept word, rare disease supplementary concept word, unique identifier, synonyms] (8393)

15 ((ectopic or extrauterin* or tubal*) adj3 pregnan* adj3 (cystectom* or resect* or enucleat* or excision* or ablation or conisation or conization or endoscop* or embol* or operation or torsion or amputation or rupture or cytoreduc*or repair or fixation or reconstruct* or surgery or surgeries or procedure* or removal)).mp. [mp=title, abstract, original title, name of substance word, subject heading word, floating sub-heading word, keyword heading word, organism supplementary concept word, protocol supplementary concept word, rare disease supplementary concept word, unique identifier, synonyms] (935)

16 ((sacrospinous or uterosacral or sacral ligament) adj3 (fixat* or suspens* or plicat*)).mp. (621)

17 ((dilatation or curettage or evacuation) adj5 (conception or cervix or cervic* or uter* or pregnancy or placent*)).mp. (5649)

18 or/4-17 (264737)

Annotation: Gyne surgery

19 ((chemoprophylax* chemoprophylactic*or prophylax* or prophylactic*) and (venous or vein or thromb*)).ti,ab. (6974)

20 (prevent* adj3 (venous or vein or thromb*)).mp. (15635)

21 (thromboprophylax* or thromboprophylactic*).mp. [mp=title, abstract, original title, name of substance word, subject heading word, floating sub-heading word, keyword heading word, organism supplementary concept word, protocol supplementary concept word, rare disease supplementary concept word, unique identifier, synonyms] (5231)

22 *Postoperative Complications/ (162066)

23 Postoperative Complications/ep, et, pc [Epidemiology, Etiology, Prevention & Control] (147345)

24 Risk Factors/ (842079)

25 (ep or ae).fs. and (venous or thromb* or bleed* or haemorr* or hemorr*).ti,ab. (210483)

26 (risk* or high-risk or incidence* or meta?analysis or analysis or complication* or outcome* or safety or versus or thrombosis or transfusion* or adverse or bleed* or haemorr* or hemorr*).ti. (2415004)

27 or/19-26 (3186398)

28 embolism/ or exp pulmonary embolism/ or exp thromboembolism/ (103748)

29 exp Thrombosis/ (130792)

30 (DVT or VTE or PE).ti,ab. (64583)

31 ((venous or vein or pulmonary or lung) adj3 (emboli* or thromb*)).mp. [mp=title, abstract, original title, name of substance word, subject heading word, floating sub-heading word, keyword heading word, organism supplementary concept word, protocol supplementary concept word, rare disease supplementary concept word, unique identifier, synonyms] (129712)

32 (DVT or VTE or PE or PTE).ti,ab. (66649)

33 or/28-32 (296625)

Annotation: VTE block

34 27 and 33 (131262)

Annotation: post op comps AND VTE

35 18 and 34 (2084)

36 animals/ not humans/ (4726677)

37 35 not 36 (2054)

38 limit 37 to ed=20191022-20201126 (88)

39 limit 37 to yr="2019 -Current" (178)

**Embase**

Database: Embase <1974 to 2020 November 25>

Search Strategy:

--------------------------------------------------------------------------------

1 exp gynecologic surgery/ or exp female genital system/su or exp gynecologic disease/su or uterine artery/su or oocyte retrieval/ (207241)

2 exp urethra/su or urinary tract/su or urogenital system/su or bladder/su or ureter/su or urothelium/su or urologic surgery/ or urinary tract surgery/ or urinary tract surgery/ or exp bladder surgery/ or exp ureter surgery/ or exp urethra surgery/ or exp urinary diversion/ or exp urine incontinence/su or urinary tract disease/su or exp bladder disease/su or exp obstructive uropathy/su or exp proteinuria/su or residual urine/su or exp ureter disease/su or exp urethra disease/su or exp urinary dysfunction/su or exp urinary tract fistula/su or exp urinary tract infection/su or exp urinary tract inflammation/su or exp urinary tract injury/su or exp urinary tract malformation/su or exp urinary tract tumor/su or exp urolithiasis/su (139818)

3 limit 2 to female (64761)

4 1 or 3 (262171)

5 ((gynecolog* or ovar* or vulv* or vagin* or adnex* or fibroid or myoma or leiomyoma or pelvic or perineal or endometri* or cervic* or cervix or uterus* or uterin* or fallop*) adj3 (resect* or excision* or ablation or conisation or conization or endoscop* or embol* or operation or torsion or amputation or rupture or cytoreduc*or repair or fixation or reconstruct* or surgery or surgeries)).mp. (99110)

6 (cervicopex* or cervicectom* or colpectom* or colporrhagh or compopex* or colposcopy* or colposuspen* or colpotom* or culdoplast* or exenteration* or fibroidectom* or fimbriectom* or hysterectomy* or hysteropex* or hysteroscop* or hysterosapingo* or LAVH or LEEP or LLETZ or myomectomy* or oophorectom* or omentectom* or ovariectom* or perineorrhaph* or sacrocolpop* or sacropex* or salpingectom* or salpingostom* or salpingo-oophorectom* or trachelectom* or tubectom* or tuboplast* or uteroscop* or vaginotom* or vulvectom* or Wertheim).mp. (178315)

7 (oocyte adj2 (retriev* or aspirat* or collect*)).mp. (10494)

8 (Tubal adj2 (excision or ligation or occlusion or ring or rings or sterilization or reanastomosis)).mp. (5472)

9 ((vaginal or retropubic or transobturator) adj3 (tape or sling)).mp. (6098)

10 (TVT or mini-sling or miniarc or bulkamid).mp. (3941)

11 (Gyneco* adj3 surg*).jw. (11995)

12 (excision adj3 loop).mp. (2047)

13 ((ovar* or luteum or luteal) adj3 (cystectom* or resect* or enucleat* or excision* or ablation or conisation or conization or endoscop* or embol* or operation or torsion or amputation or rupture or cytoreduc*or repair or fixation or reconstruct* or surgery or surgeries or procedure* or removal)).mp. (13213)

14 ((ectopic or extrauterin* or tubal*) adj3 pregnan* adj3 (cystectom* or resect* or enucleat* or excision* or ablation or conisation or conization or endoscop* or embol* or operation or torsion or amputation or rupture or cytoreduc*or repair or fixation or reconstruct* or surgery or surgeries or procedure* or removal)).mp. (1171)

15 ((sacrospinous or uterosacral or sacral ligament) adj3 (fixat* or suspens* or plicat*)).mp. (1604)

16 ((dilatation or curettage or evacuation) adj5 (conception or cervix or cervic* or uter* or pregnancy or placent*)).mp. (9635)

17 or/4-16 (372731)

18 ((chemoprophylax* or chemoprophylactic* or prophylax* or prophylactic*) and (venous or vein or thromb*)).ti,ab. (32406)

19 (prevent* adj3 (venous or vein or thromb*)).mp. (31684)

20 (thromboprophylax* or thromboprophylactic*).mp. (8598)

21 *postoperative complication/co, ep, et, pc (38447)

22 exp *venous thromboembolism/co, ep, et, pc (19638)

23 thrombosis prevention/ (12041)

24 postoperative complication/ep (10605)

25 *venous thromboembolism/ (16948)

26 *deep vein thrombosis/ (17391)

27 venous thromboembolism/ep (1539)

28 risk factor/ (1077450)

29 (ep or co).fs. and (venous or thromb* or bleed* or haemorr* or hemorr*).ti,ab. (235619)

30 (risk* or high-risk or incidence* or meta?analysis or analysis or complication* or outcome* or safety or versus or thrombosis or transfusion* or adverse or bleed* or haemorr* or hemorr*).ti. (3163867)

31 or/18-30 (4047180)

Annotation: post op VTE comp

32 exp thromboembolism/ (493649)

33 (DVT or VTE or PE or PTE).ti,ab. (98133)

34 ((venous or vein or pulmonary or lung) adj3 (emboli* or thromb*)).mp. (245822)

35 or/32-34 (570078)

Annotation: VTE Broad

36 31 and 35 (254589)

Annotation: risk of post-op VTE

37 17 and 36 (5747)

Annotation: gyne surgery and risk of VTE

38 exp animals/ or exp invertebrate/ or animal experiment/ or animal model/ or animal tissue/ or animal cell/ or nonhuman/ (28384151)

39 human/ or normal human/ or human cell/ (21828908)

40 38 and 39 (21762932)

41 38 not 40 (6621219)

42 37 not 41 (5704)

43 exp controlled clinical trial/ (824320)

44 42 not 43 (5443)

45 clinical study/ (156593)

46 case control study/ (164662)

47 family study/ (26197)

48 longitudinal study/ (148411)

49 retrospective study/ (997612)

50 prospective study/ (645917)

51 cohort analysis/ (641833)

52 (Cohort adj (study or studies)).mp. (327129)

53 (Case control adj (study or studies)).tw. (139098)

54 (follow up adj (study or studies)).tw. (64966)

55 (observational adj (study or studies)).tw. (178280)

56 (epidemiologic$ adj (study or studies)).tw. (109159)

57 (cross sectional adj (study or studies)).tw. (234876)

58 or/45-57 (2881708)

59 44 and 58 (1778)

60 (prognosis or prognostic or predict* or risk*).mp. (6407373)

61 (incidence* or outcome* or comparison* or complication*).ti. (1254524)

62 prevalence.mp. or prevalence/ (1103246)

63 baseline.mp. (989522)

64 or/60-63 (8278158)

65 44 and 64 (3838)

66 59 or 65 (4129)

67 limit 66 to em=201940-202052 (410)

68 limit 66 to yr="2019 -Current" (644)

69 67 or 68 (693)

**Web of Science**

# 27 [**583**](https://apps.webofknowledge.com/summary.do?product=WOS&doc=1&qid=29&SID=8DKczHLYjV3blWM1aaC&search_mode=AdvancedSearch&update_back2search_link_param=yes) #26

*Indexes=SCI-EXPANDED, SSCI, A&HCI, CPCI-S, CPCI-SSH, ESCI Timespan=2019-2020*

# 26 [**4,485**](https://apps.webofknowledge.com/summary.do?product=WOS&doc=1&qid=28&SID=8DKczHLYjV3blWM1aaC&search_mode=CombineSearches&update_back2search_link_param=yes) #25 AND #9

*Indexes=SCI-EXPANDED, SSCI, A&HCI, CPCI-S, CPCI-SSH, ESCI Timespan=1976-2020*

# 25 [**159,247**](https://apps.webofknowledge.com/summary.do?product=WOS&doc=1&qid=27&SID=8DKczHLYjV3blWM1aaC&search_mode=CombineSearches&update_back2search_link_param=yes) #24 OR #23 OR #22 OR #21 OR #20 OR #19 OR #18 OR #17 OR #16 OR #15 OR #14 OR #13 OR #12 OR #11 OR #10

*Indexes=SCI-EXPANDED, SSCI, A&HCI, CPCI-S, CPCI-SSH, ESCI Timespan=1976-2020*

# 24 [**3,711**](https://apps.webofknowledge.com/summary.do?product=WOS&doc=1&qid=26&SID=8DKczHLYjV3blWM1aaC&search_mode=AdvancedSearch&update_back2search_link_param=yes) TS=((dilatation or curettage or evacuation) near/5 (conception or cervix or cervic* or uter* or pregnancy or placent*) )

*Indexes=SCI-EXPANDED, SSCI, A&HCI, CPCI-S, CPCI-SSH, ESCI Timespan=1976-2020*

# 23 [**1,098**](https://apps.webofknowledge.com/summary.do?product=WOS&doc=1&qid=25&SID=8DKczHLYjV3blWM1aaC&search_mode=AdvancedSearch&update_back2search_link_param=yes) TS=((sacrospinous or uterosacral or sacral) NEAR/3 (fixat* or suspens* or plicat*) )

*Indexes=SCI-EXPANDED, SSCI, A&HCI, CPCI-S, CPCI-SSH, ESCI Timespan=1976-2020*

# 22 [**496**](https://apps.webofknowledge.com/summary.do?product=WOS&doc=1&qid=24&SID=8DKczHLYjV3blWM1aaC&search_mode=AdvancedSearch&update_back2search_link_param=yes) TS=(((ectopic or extrauterin* or tubal*) NEAR/3 (pregnan*) NEAR/3 (resect* or excision* or ablation or repair or fixation or reconstruct* or surger* or procedure* or removal) ))

*Indexes=SCI-EXPANDED, SSCI, A&HCI, CPCI-S, CPCI-SSH, ESCI Timespan=1976-2020*

# 21 [**109**](https://apps.webofknowledge.com/summary.do?product=WOS&doc=1&qid=23&SID=8DKczHLYjV3blWM1aaC&search_mode=AdvancedSearch&update_back2search_link_param=yes) TS=((luteum or luteal) NEAR/3 (resect* or excision* or ablation or coni?ation or endoscop* or embol* or repair or fixation or reconstruct* or surger* or procedure* or removal) )

*Indexes=SCI-EXPANDED, SSCI, A&HCI, CPCI-S, CPCI-SSH, ESCI Timespan=1976-2020*

# 20 [**1,992**](https://apps.webofknowledge.com/summary.do?product=WOS&doc=1&qid=22&SID=8DKczHLYjV3blWM1aaC&search_mode=AdvancedSearch&update_back2search_link_param=yes) TS=((fallop* or curettage or labia* or clitoris) NEAR/3 (resect* or excision* or ablation or coni?ation or endoscop* or embol* or repair or fixation or reconstruct* or surger* or procedure* or removal) )

*Indexes=SCI-EXPANDED, SSCI, A&HCI, CPCI-S, CPCI-SSH, ESCI Timespan=1976-2020*

# 19 [**23,062**](https://apps.webofknowledge.com/summary.do?product=WOS&doc=1&qid=21&SID=8DKczHLYjV3blWM1aaC&search_mode=AdvancedSearch&update_back2search_link_param=yes) TS=((endometri* or cervic* or cervix or uterus or uterin*) NEAR/3 (resect* or excision* or ablation or coni?ation or endoscop* or embol* or repair or fixation or reconstruct* or surger* or procedure* or removal) )

*Indexes=SCI-EXPANDED, SSCI, A&HCI, CPCI-S, CPCI-SSH, ESCI Timespan=1976-2020*

# 18 [**15,588**](https://apps.webofknowledge.com/summary.do?product=WOS&doc=1&qid=20&SID=8DKczHLYjV3blWM1aaC&search_mode=AdvancedSearch&update_back2search_link_param=yes) TS=((fibroid* or myoma or leiomyoma or pelvic or perineal) NEAR/3 (resect* or excision* or ablation or coni?ation or endoscop* or embol* or repair or fixation or reconstruct* or surger* or procedure* or removal) )

*Indexes=SCI-EXPANDED, SSCI, A&HCI, CPCI-S, CPCI-SSH, ESCI Timespan=1976-2020*

# 17 [**16,086**](https://apps.webofknowledge.com/summary.do?product=WOS&doc=1&qid=19&SID=8DKczHLYjV3blWM1aaC&search_mode=AdvancedSearch&update_back2search_link_param=yes) TS=((ovar* or vulv* or vagin* or transvagin* or adnex*) NEAR/3 (resect* or excision* or ablation or coni?ation or endoscop* or embol* or repair or fixation or reconstruct* or surger* or procedure* or removal) )

*Indexes=SCI-EXPANDED, SSCI, A&HCI, CPCI-S, CPCI-SSH, ESCI Timespan=1976-2020*

# 16 [**1,620**](https://apps.webofknowledge.com/summary.do?product=WOS&doc=1&qid=18&SID=8DKczHLYjV3blWM1aaC&search_mode=AdvancedSearch&update_back2search_link_param=yes) TS=(excision near/3 loop)

*Indexes=SCI-EXPANDED, SSCI, A&HCI, CPCI-S, CPCI-SSH, ESCI Timespan=1976-2020*

# 15 [**2,743**](https://apps.webofknowledge.com/summary.do?product=WOS&doc=1&qid=16&SID=8DKczHLYjV3blWM1aaC&search_mode=AdvancedSearch&update_back2search_link_param=yes) TS=(TVT or mini-sling or miniarc or bulkamid)

*Indexes=SCI-EXPANDED, SSCI, A&HCI, CPCI-S, CPCI-SSH, ESCI Timespan=1976-2020*

# 14 [**6,370**](https://apps.webofknowledge.com/summary.do?product=WOS&doc=1&qid=15&SID=8DKczHLYjV3blWM1aaC&search_mode=AdvancedSearch&update_back2search_link_param=yes) TS=((vaginal or pubovaginal or transvaginal or retropubic or transobturator or prolapse) near/3 (tape* or sling* or mesh*) )

*Indexes=SCI-EXPANDED, SSCI, A&HCI, CPCI-S, CPCI-SSH, ESCI Timespan=1976-2020*

# 13 [**3,317**](https://apps.webofknowledge.com/summary.do?product=WOS&doc=1&qid=14&SID=8DKczHLYjV3blWM1aaC&search_mode=AdvancedSearch&update_back2search_link_param=yes) TS=(Tubal near/2 (excision or ligation or occlusion or ring or rings or sterilization or reanastomosis) )

*Indexes=SCI-EXPANDED, SSCI, A&HCI, CPCI-S, CPCI-SSH, ESCI Timespan=1976-2020*

# 12 [**8,387**](https://apps.webofknowledge.com/summary.do?product=WOS&doc=1&qid=13&SID=8DKczHLYjV3blWM1aaC&search_mode=AdvancedSearch&update_back2search_link_param=yes) TS=(oocyte* near/2 (retriev* or aspirat* or collect*) )

*Indexes=SCI-EXPANDED, SSCI, A&HCI, CPCI-S, CPCI-SSH, ESCI Timespan=1976-2020*

# 11 [**97,909**](https://apps.webofknowledge.com/summary.do?product=WOS&doc=1&qid=11&SID=8DKczHLYjV3blWM1aaC&search_mode=AdvancedSearch&update_back2search_link_param=yes) TS=(cervicopex* or cervicectom* or colpectom* or colporrhaph or colpopex* or colposcop* or colposuspen* or colpotom* or culdoplast* or exenteration* or fibroidectom* or fimbriectom* or hysterectom* or hysteropex* or hysteroscop* or hysterosalpingo* or LAVH or LEEP or LLETZ or USLS or myomectom* or oophorectom* or omentectom* or ovariectom* or perineoplast* or perineorrhaph* or sacrocolpop* or sacropex* or salpingectom* or salpingostom* or salpingo-oophorectom* or trachelectom* or tubectom* or tuboplast* or uteroscop* or vaginotom* or vaginoplast* or vulvectom* or Wertheim or labiaplast*)

*Indexes=SCI-EXPANDED, SSCI, A&HCI, CPCI-S, CPCI-SSH, ESCI Timespan=1976-2020*

# 10 [**8,841**](https://apps.webofknowledge.com/summary.do?product=WOS&doc=1&qid=10&SID=8DKczHLYjV3blWM1aaC&search_mode=AdvancedSearch&update_back2search_link_param=yes) TS=(((gyn$ecolog* or gynecol* or gynaecol*) NEAR/3 (surgery or surgical or surgeries) ))

*Indexes=SCI-EXPANDED, SSCI, A&HCI, CPCI-S, CPCI-SSH, ESCI Timespan=1976-2020*

# 9 [**785,175**](https://apps.webofknowledge.com/summary.do?product=WOS&doc=1&qid=9&SID=8DKczHLYjV3blWM1aaC&search_mode=CombineSearches&update_back2search_link_param=yes) #8 AND #3

*Indexes=SCI-EXPANDED, SSCI, A&HCI, CPCI-S, CPCI-SSH, ESCI Timespan=1976-2020*

# 8 [**5,265,213**](https://apps.webofknowledge.com/summary.do?product=WOS&doc=1&qid=8&SID=8DKczHLYjV3blWM1aaC&search_mode=CombineSearches&update_back2search_link_param=yes) #7 OR #6 OR #5 OR #4

*Indexes=SCI-EXPANDED, SSCI, A&HCI, CPCI-S, CPCI-SSH, ESCI Timespan=1976-2020*

# 7 [**4,927,470**](https://apps.webofknowledge.com/summary.do?product=WOS&doc=1&qid=7&SID=8DKczHLYjV3blWM1aaC&search_mode=AdvancedSearch&update_back2search_link_param=yes) TS=(complication* or outcome* or safety or versus or thrombosis or transfusion* or adverse or bleed* or haemorr* or hemorr*)

*Indexes=SCI-EXPANDED, SSCI, A&HCI, CPCI-S, CPCI-SSH, ESCI Timespan=1976-2020*

# 6 [**6,870**](https://apps.webofknowledge.com/summary.do?product=WOS&doc=1&qid=6&SID=8DKczHLYjV3blWM1aaC&search_mode=AdvancedSearch&update_back2search_link_param=yes) TS=(thromboprophylax* or thromboprophylactic*)

*Indexes=SCI-EXPANDED, SSCI, A&HCI, CPCI-S, CPCI-SSH, ESCI Timespan=1976-2020*

# 5 [**636,352**](https://apps.webofknowledge.com/summary.do?product=WOS&doc=1&qid=5&SID=8DKczHLYjV3blWM1aaC&search_mode=AdvancedSearch&update_back2search_link_param=yes) TS=(prevent* NEAR/3 venous or vein or thromb*)

*Indexes=SCI-EXPANDED, SSCI, A&HCI, CPCI-S, CPCI-SSH, ESCI Timespan=1976-2020*

# 4 [**6,651**](https://apps.webofknowledge.com/summary.do?product=WOS&doc=1&qid=4&SID=8DKczHLYjV3blWM1aaC&search_mode=AdvancedSearch&update_back2search_link_param=yes) TS=((chemoprophylax* or chemoprophylactic*or prophylax* or prophylactic*) and (venous or vein or thromb*) )

*Indexes=SCI-EXPANDED, SSCI, A&HCI, CPCI-S, CPCI-SSH, ESCI Timespan=1976-2020*

# 3 [**1,217,228**](https://apps.webofknowledge.com/summary.do?product=WOS&doc=1&qid=3&SID=8DKczHLYjV3blWM1aaC&search_mode=CombineSearches&update_back2search_link_param=yes) #2 OR #1

*Indexes=SCI-EXPANDED, SSCI, A&HCI, CPCI-S, CPCI-SSH, ESCI Timespan=1976-2020*

# 2 [**1,202,436**](https://apps.webofknowledge.com/summary.do?product=WOS&doc=1&qid=2&SID=8DKczHLYjV3blWM1aaC&search_mode=AdvancedSearch&update_back2search_link_param=yes) TS=(venous or vein or pulmonary or lung NEAR/3 emboli* or thromb*)

*Indexes=SCI-EXPANDED, SSCI, A&HCI, CPCI-S, CPCI-SSH, ESCI Timespan=1976-2020*

# 1 [**33,743**](https://apps.webofknowledge.com/summary.do?product=WOS&doc=1&qid=1&SID=8DKczHLYjV3blWM1aaC&search_mode=AdvancedSearch&update_back2search_link_param=yes) TS=(DVT or VTE or PE or PTE)

*Indexes=SCI-EXPANDED, SSCI, A&HCI, CPCI-S, CPCI-SSH, ESCI Timespan=1976-2020*

**Google Scholar**

Query Google Scholar using Publish or Perish macOS Version: 7.27.2949 (2 October 2020)

Harzing, A.W. (2007) Publish or Perish, available from <https://harzing.com/resources/publish-or-perish>

Search terms

Keywords: thromboembolism AND incidence AND (surgery OR resection OR excision OR operation) AND (gynecology or gynaecology)

Years: all

Years: 2019 to 2020

**Supplementary Methods.** Further details regarding study selection

Through discussion and consensus building, expert panelists (including experienced general abdominal and gynecological surgeons and clinician-methodologists) selected the most relevant general abdominal and gynecological procedures for this study. We included observational studies that enrolled a minimum of 50 adult patients undergoing a target procedure in general abdominal or gynecological surgery that reported the incidence of at least one of the patient-important outcomes of interest: fatal PE, symptomatic PE, symptomatic DVT, symptomatic VTE, fatal bleeding, and major bleeding (bleeding requiring re-operation, transfusion, or post-operative hemoglobin below 70 g/L).

We also excluded high risk of bias articles, when enough studies with low risk of bias are identified for a target procedure:

If we had at least 1,000 patients and five articles for a target procedure in studies with very low and low RoB, we excluded studies with moderate and high RoB. If we had at least 2,000 patients and 10 articles for a certain target procedure in studies with very low, low or moderate RoB, we excluded studies with high RoB. In other situations, we used all studies irrespective of their RoB.

In case of overlapping study populations, we primarily included the article with lowest risk of bias and secondarily the article with greater number of patients.

Finally, from these studies, we included all those who provided (some) information on thromboprophylaxis.

**Appendix S1. The Risk of Thrombosis and Bleeding in General and Gynecologic Surgery (ROTBIGGS) Investigators**

Besides authors of this Research Letter, the ROTBIGGS Investigators include following researchers: Riikka L. Aaltonen, MD, PhD, Kirsi M. Joronen, MD, PhD (both from the Turku University Hospital and University of Turku, Turku, Finland); Karoliina M. Aro, MD, PhD, Ines Beilmann-Lehtonen, MD, Päivi J. Galambosi, MD, PhD, Ilkka E. J. Kalliala, MD, PhD, Tuomas P. Kilpeläinen, MD, PhD, Antti J. Kivelä, MD, PhD, Hanna Lampela, MD, PhD, Anna Luomaranta, MD, PhD, Ville J. Sallinen, MD, PhD (all from the University of Helsinki and Helsinki University Hospital, Helsinki, Finland); Marco H. Blanker, MD, PhD (University Medical Center Groningen, University of Groningen, Groningen, The Netherlands); Joviata L. Cardenas, MD, PhD (National Center for Health Technology Excellence (CENETEC) Direction of Health Technologies assessment, Mexico City, Mexico); Jaana Elberkennou, MD (Vaasa Central Hospital, Vaasa, Finland); Herney A. Garcia-Perdomo, MD, PhD (Universidad del Valle, Cali, Colombia); Fang Zhou Ge, BSc, Yung Lee, MD, Borna Tadayon Najafabadi, MD, MPH, Yuting Wang, MD, Yingqi Xiao, MSc, Liang Yao, MSc (all from McMaster University, Hamilton, Canada); Huda Gomaa, MSc (Alexandria University, Alexandria, Egypt); Matthew L. Izett-Kay, BM BS (The John Radcliffe Hospital, Oxford University Hospitals, Oxford, UK); Paul J. Karanicolas, MD, PhD (Sunnybrook Health Sciences Centre and University of Toronto, Toronto, Canada); Päivi K. Karjalainen, MD, PhD, Anne K. Mattila, MD, PhD (both from the Central Finland Central Hospital, Jyväskylä, Finland); Nadina Khamani, MD, PhD (Sechenov University, Moscow, Russia); Taina P. Nykänen, MD, PhD (Hyvinkää Hospital, Hyvinkää, Finland); Sanna M. Oksjoki, MD, PhD (Felicitas Mehiläinen Turku, Turku, Finland); Sanjay Pandanaboyana, MS, Mphil (Freeman Hospital and Newcastle University, Newcastle Upon Tyne, UK); Chathura B. B. Ratnayake, MBChB (The University of Auckland and Auckland City Hospital, Auckland, New Zealand); Aleksi R. Raudasoja, MD (University of Helsinki, Helsinki, Finland); Tino Singh, BM (University of Eastern Finland, Kuopio, Finland); Riikka M. Tähtinen, MD, PhD (Tampere University Hospital, Tampere, Finland); Robin W. M. Vernooij, MD, PhD (University Medical Center Utrecht and Utrecht University, Utrecht, The Netherlands).

**Figure S1. Study flow chart (general surgery)**


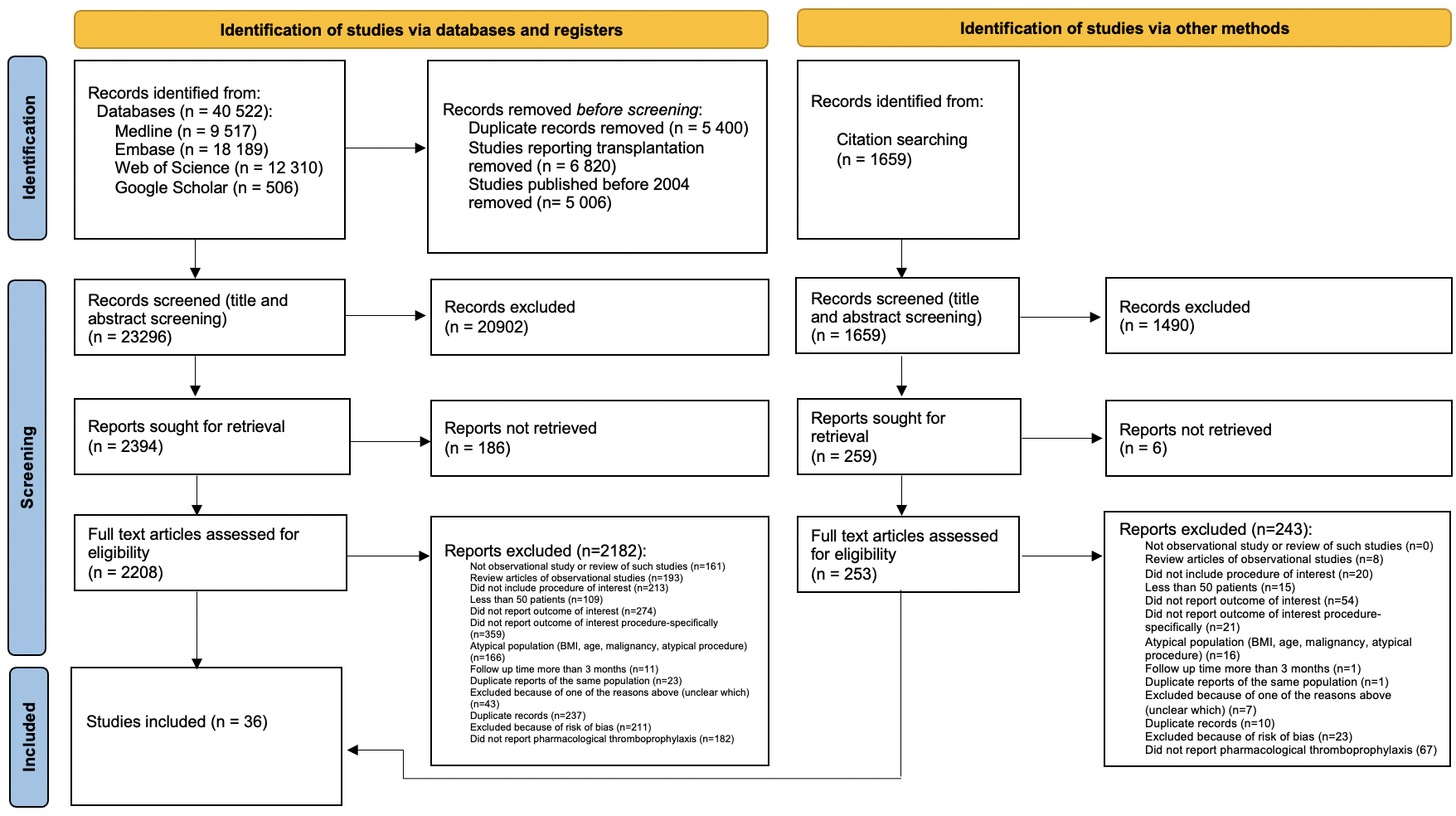


**Figure S2. Study flow chart (gynecological surgery)**


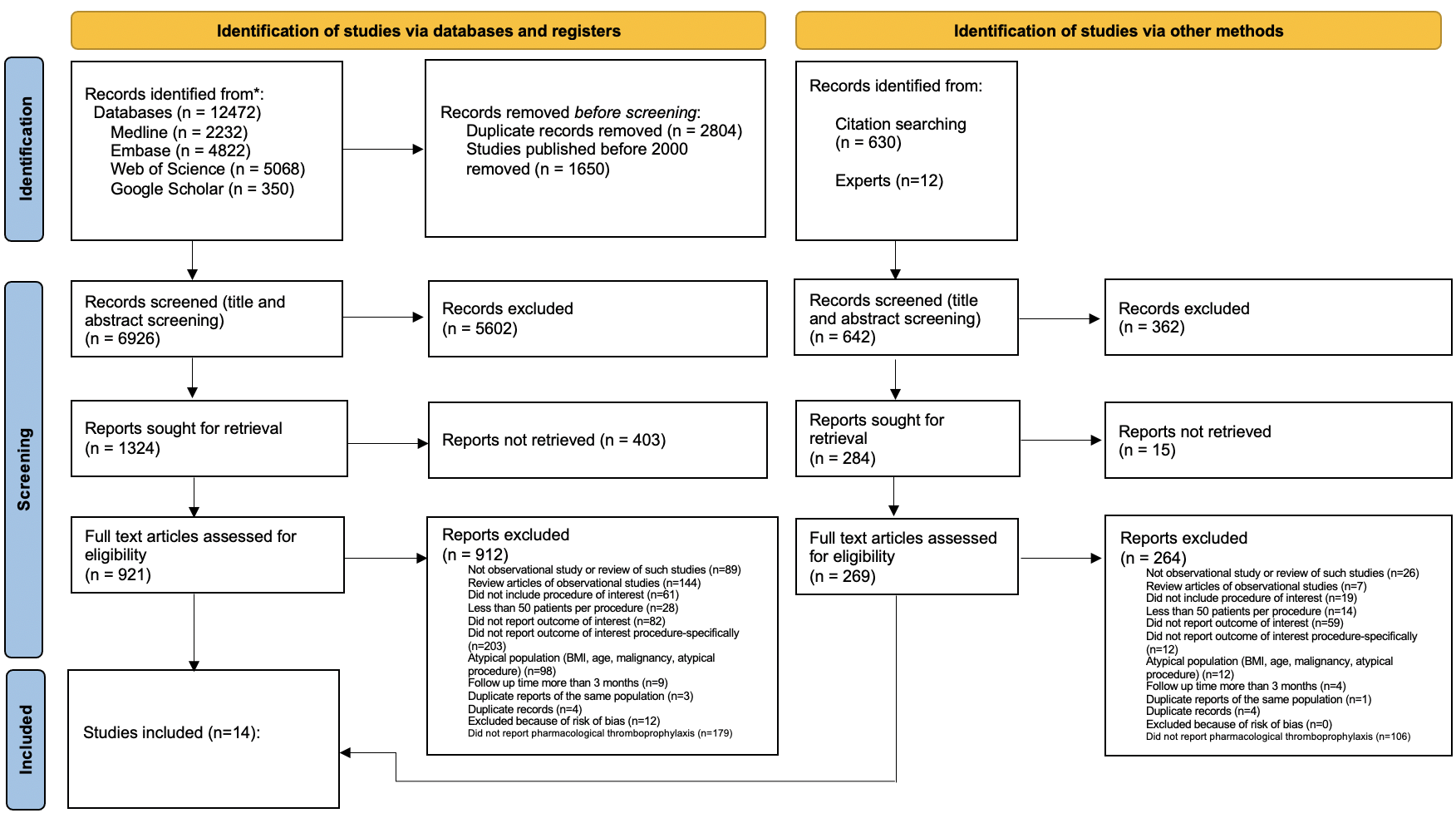


**Table S1. List of the included 50 studies, stratified by procedure (in chronological order)**

| **Procedure** | **Citation** | |
| --- | --- | --- |
| **Laparoscopic cholecystectomy** | | |
|  | Gundogdu RH, Oduncu M, Bozkirli BO, Yazicioglu MO, Akbaba S. Does thromboprophylaxis cause bleeding after laparoscopic cholecystectomy? Bratisl Lek Listy 2017;118(3):156-159. | |
|  | Ulrych J, Kvasnicka T, Fryba V, et al. 28 day post-operative persisted hypercoagulability after surgery for benign diseases: a prospective cohort study. BMC Surg 2016;16:16. | |
|  | Pakaneh MA, Pazouki A, Tamannaie Z, Hakimian M, Zohrei HR, Chaichian S. Results of post-laparoscopic cholecystectomy duplex scan without deep vein thrombosis prophylaxis prior to surgery. Med J Islam Repub Iran 2012;26(4):164-6. | |
|  | Ntourakis D, Sergentanis TN, Georgiopoulos I, et al. Subclinical activation of coagulation and fibrinolysis in laparoscopic cholecystectomy: do risk factors exist? Int J Surg 2011;9(5):374-7. | |
|  | Triantafyllidis I, Nikoloudis N, Sapidis N, Chrissidou M, Kalaitsidou I, Chrissidis T. Complications of laparoscopic cholecystectomy: our experience in a district general hospital. Surg Laparosc Endosc Percutan Tech 2009;19(6):449-58. | |
|  | Rathore MA, Andrabi SI, Mansha M, Brown MG. Day case laparoscopic cholecystectomy is safe and feasible: a case controlled study. Int J Surg 2007;5(4):255-9. | |
|  | Lindberg F, Björck M, Rasmussen I, Nyman R, Bergqvist D. Low frequency of phlebographic deep vein thrombosis after laparoscopic cholecystectomy--a pilot study. Clin Appl Thromb Hemost 2006;12(4):421-6. | |
| **Laparoscopic sleeve gastrectomy** | | |
|  | Abuoglu HH, Müftüoğlu MAT, Odabaşı M. A new protocol for venous thromboembolism prophylaxis in bariatricsurgery. Obes Surg 2019;29(2):729-734. | |
|  | AlKhaldi LK, AlSaffar NA, AlHamdan F, et al. Long-term outcomes after laparoscopic sleeve gastrectomy in Kuwait. Ann Saudi Med 2019;39(2):100-103. | |
|  | Nimeri AA, Bautista J, Ibrahim M, et al. Mandatory risk assessment reduces venous thromboembolism in bariatric surgery patients. Obes Surg 2018;28(2):541-547. | |
|  | Brunetti L, Wassef A, Sadek R, et al. Anticoagulant activity of enoxaparin and unfractionated heparin for venous thromboembolism prophylaxis in obese patients undergoing sleeve gastrectomy. Surg Obes Relat Dis 2019;15(3):363-373. | |
|  | Thereaux J, Lesuffleur T, Czernichow S, et al. To what extent does posthospital discharge chemoprophylaxis prevent venous thromboembolism after bariatric surgery?: Results from a nationwide cohort of more than 110,000 patients. Ann Surg 2018;267(4):727-733. | |
|  | Moradian S, Daneshpajouh A, Patel A, Kitzman K, Gomez N, Choi M. Laparoscopic sleeve gastrectomy without over-sewing the staple line: A case series demonstrating efficacy and minimization of both intra- and post-operative complications. Int J Surg Open 2017;8:7-10. | |
|  | Sakran N, Raziel A, Goitein O, Szold A, Goitein D. Laparoscopic sleeve gastrectomy for morbid obesity in 3003 patients: results at a high-volume bariatric center. Obes Surg 2016;26(9):2045-2050. | |
|  | Alsina E, Ruiz-Tovar J, Alpera MR, et al. Incidence of deep vein thrombosis and thrombosis of the portal-mesenteric axis after laparoscopic sleeve gastrectomy. J Laparoendosc Adv Surg Tech A 2014;24(9):601-5. | |
|  | Biertho L, Lebel S, Marceau S, et al. Laparoscopic sleeve gastrectomy: with or without duodenal switch? A consecutive series of 800 cases. Dig Surg 2014;31(1):48-54. | |
|  | Woo HD, Kim YJ. Prevention of venous thromboembolism with enoxaparin in bariatirc surgery. J Korean Surg Soc 2013;84(5):298-303. | |
| **Open gastric bypass** | | |
|  | Santo MA, Pajecki D, Riccioppo D, Cleva R, Kawamoto F, Cecconello I. Early complications in bariatric surgery: incidence, diagnosis and treatment. Arq Gastroenterol 2013;50(1):50-5. | |
|  | Slotman GJ. Non-transectional open gastric bypass as the definitive bariatric procedure for 61 patients with BMI of 70 and higher. Obes Surg 2010;20(1):7-12. | |
|  | Caruana JA, Anain PM, Pham DT. The pulmonary embolism risk score system reduces the incidence and mortality of pulmonary embolism after gastric bypass. Surgery 2009;146(4):678-83; discussion 683-5. | |
|  | Gargiulo NJ 3rd, Veith FJ, Lipsitz EC, et al. The incidence of pulmonary embolism in open versus laparoscopic gastric bypass. Ann Vasc Surg 2007;21(5):556-9. | |
|  | Abou-Nukta F, Alkhoury F, Arroyo K, et al. Clinical pulmonary embolus after gastric bypass surgery. Surg Obes Relat Dis 2006;2(1):24-8; discussion 29. | |
|  | Gargiulo NJ 3rd, Veith FJ, Lipsitz EC, Suggs WD, Ohki T, Goodman E. Experience with inferior vena cava filter placement in patients undergoing open gastric bypass procedures. J Vasc Surg 2006;44(6):1301-5. | |
|  | Cotter SA, Cantrell W, Fisher B, Shopnick R. Efficacy of venous thromboembolism prophylaxis in morbidly obese patients undergoing gastric bypass surgery. Obes Surg 2005;15(9):1316-20. | |
| **Open groin hernia repair** | | |
|  | Poudel S, Miyazaki K, Hirano S. Continuation of antithrombotic therapy increases minor bleeding but does not increase the risk other morbidities in open inguinal hernia repair: A propensity score-matched analysis. Hernia 2020;24(4):857-865. | |
|  | Wang M, Zhang G, Chen J, et al. Current prevalence of perioperative early venous thromboembolism and risk factors in Chinese adult patients with inguinal hernia (CHAT-1). Sci Rep 2020;10(1):12667. | |
|  | Lozano FS, Sánchez-Fernández J, González-Porras JR, et al. Slow femoral venous flow and venous thromboembolism following inguinal hernioplasty in patients without or with low molecular weight heparin prophylaxis. Hernia 2015;19(6):901-8. | |
|  | Bessa SS, Abdel-fattah MR, Al-Sayes IA, Korayem IT. Results of prosthetic mesh repair in the emergency management of the acutely incarcerated and/or strangulated groin hernias: a 10-year study. Hernia 2015;19(6):909-14. | |
|  | Srsen D, Druzijanić N, Pogorelić Z, et al. Quality of life analysis after open and laparoscopic inguinal hernia repair--retrospective study. Hepatogastroenterology 2008;55(88):2112-5. | |
|  | Holzheimer RG. Low recurrence rate in hernia repair--results in 300 patients with open mesh repair of primary inguinal hernia. Eur J Med Res. 2007;12(1):1-5. | |
| **Open liver resection** | | |
|  | Ainoa E, Uutela A, Nordin A, Mäkisalo H, Sallinen V. Pre- vs. postoperative initiation of thromboprophylaxis in liver surgery. HPB (Oxford) 2021;23(7):1016-1024. | |
|  | Kron P, Kimura N, Farid S, Lodge JPA. Current role of trisectionectomy for hepatopancreatobiliary malignancies. Ann Gastroenterol Surg 2019;3(6):606-619. | |
|  | Singh SA, Vivekananthan P, Sharma A, Sharma S, Bharathy KG. Retrospective analysis of post-operative coagulopathy after major hepatic resection at a tertiary care centre in Northern India. Indian J Anaesth. 2017;61(7):575-580. | |
|  | Nathan H, Weiss MJ, Soff GA, at el. Pharmacologic prophylaxis, postoperative INR, and risk of venous thromboembolism after hepatectomy. J Gastrointest Surg 2014;18(2):295-302; discussion 302-3. | |
|  | Aramaki O, Takayama T, Higaki T, et al. Decreased blood loss reduces postoperative complications in resection for hepatocellular carcinoma. J Hepatobiliary Pancreat Sci 2014;21(8):585-91. | |
|  | Barbas AS, Turley RS, Mallipeddi MK, et al. Examining reoperation and readmission after hepatic surgery. J Am Coll Surg. 2013;216(5):915-23. | |
| **Open radical hysterectomy** | | |
|  | Neagoe OC, Ionica M, Mazilu O. The role of pelvic lymphocele in the development of early postoperative complications. Medicine (Baltimore) 2018;97(37):e12353. | |
|  | Thomas A, Chandy R, Sebastian A, et al. Surgical outcomes and patterns of recurrence in endometrial cancers. J Gynecol Surg 2017;33(3):97-104. | |
|  | Gao H, Zhang Z. Laparoscopy Versus Laparotomy in the Treatment of High-Risk Endometrial Cancer: A Propensity Score Matching Analysis. Medicine (Baltimore) 2015;94(30):e1245. | |
|  | Đurđević S, Stojanović S, Pantelić M, et al. Radical hysterectomy in surgical treatment of invasive cervical cancer at the department of gynecology and obstetrics in Novi Sad in the period 1993-2013. Med Pregl 2015;68(7-8):227-233. | |
|  | Satoh T, Matsumoto K, Tanaka YO, et al. Incidence of venous thromboembolism before treatment in cervical cancer and the impact of management on venous thromboembolism after commencement of treatment. Thromb Res 2013;131(4):e127-32. | |
| Open ovarian cancer surgery | | |
|  | Heus C, Smorenburg A, Stoker J, Rutten MJ, Amant FCH, van Lonkhuijzen LRCW. Visceral obesity and muscle mass determined by CT scan and surgical outcome in patients with advanced ovarian cancer. A retrospective cohort study. Gynecol Oncol 2021;160(1):187-192. |  |
|  | Wagner BE, Langstraat CL, McGree ME, et al. Beyond prophylaxis: Extended risk of venous thromboembolism following primary debulking surgery for ovarian cancer. Gynecol Oncol 2019;152(2):286-292. | |
|  | Lu Q, Qu H, Liu C, Wang S, Zhang Z, Zhang Z. Comparison of Laparoscopy and Laparotomy in Surgical Staging of Apparent Early Ovarian Cancer: 13-year Experience. Medicine (Baltimore) 2016;95(20):e3655. | |
|  | Plotti F, Montera R, Aloisi A, et al. Total rectosigmoidectomy versus partial rectal resection in primary debulking surgery for advanced ovarian cancer. Eur J Surg Oncol. 2016 Mar;42(3):383-90. | |
|  | Fotopoulou C, Jones BP, Savvatis K, et al. Maximal effort cytoreductive surgery for disseminated ovarian cancer in a UK setting: challenges and possibilities. Arch Gynecol Obstet 2016;294(3):607-14. | |
|  | Krasiński Z, Szpurek D, Staniszewski R, et al. The value of extended preoperative thromboprophylaxis with dalteparin in patients with ovarian cancer qualified to surgical treatment. Int Angiol 2014;33(4):365-71. | |
|  | Abu Saadeh F, Norris L, O'Toole S, Gleeson N. Venous thromboembolism in ovarian cancer: incidence, risk factors and impact on survival. Eur J Obstet Gynecol Reprod Biol 2013;170(1):214-8. | |
|  | Woelber L, Jung S, Eulenburg C, et al. Perioperative morbidity and outcome of secondary cytoreduction for recurrent epithelial ovarian cancer. Eur J Surg Oncol 2010;36(6):583-8. | |
|  | Mourton SM, Temple LK, Abu-Rustum NR, et al. Morbidity of rectosigmoid resection and primary anastomosis in patients undergoing primary cytoreductive surgery for advanced epithelial ovarian cancer. Gynecol Oncol 2005;99(3):608-14. | |

**Table S2. Summary of included studies by procedure**

| **Procedure** | **Studies** | **Patients** | **Age**  **(median, years)** | **Proportion of women**  **(median, %)** | **Proportion of cancer**  **(median, %)** |
| --- | --- | --- | --- | --- | --- |
|  |  |  |  |  |  |
| **Laparoscopic cholecystectomy** | 7 | 3 017 | 49 | 76 | 0 |
| **Laparoscopic sleeve gastrectomy** | 10 | 67 001 | 68 | 40 | 0 |
| **Open gastric bypass** | 7 | 5 576 | 79 | 41 | 0 |
| **Open groin hernia repair** | 6 | 12 532 | 57 | 14 | 0 |
| **Open liver resection** | 6 | 4 692 | 61 | 42 | 89 |
| **Open radical hysterectomy** | 5 | 2 403 | 55 | 100 | 100 |
| **Open ovarian cancer surgery** | 9 | 2 137 | 59 | 100 | 100 |

**Table S3.** **Design features used for assessment of risk of bias**

| **Domain** | **Low risk of bias** | **High risk of bias** |
| --- | --- | --- |
| **Sampling** | Consecutive patient recruitment or administrative database with random sampling | Non-consecutive patient recruitment or administrative database with non-random sampling |
| **Source of information** | Prospective data collection by study investigators  Retrospective duplicate chart reviews with good documentation of agreement between reviewers | Retrospective duplicate chart reviews without documentation of agreement between reviewers  Administrative database information |
| **Recruitment years** | Studies with the majority of patient recruitment years 2010 or after | Studies with the majority of patient recruitment years 2009 or before |
| **Specification of length of follow-up** | Studies that clearly define the time period of follow-up (up to 3 months) | Studies that do not clearly define the time period of follow-up |
| **Study type** | International multicenter; Multicenter in one country; Single center, not single surgeon | Single surgeon series |
| **Overall risk of bias** | No or one high risk of bias domain: Low risk of bias  Two high risk of bias domains: Moderate risk of bias  Three or more high risk of bias domains: High risk of bias | |
|  |  | |

**Table S4.** **Risk of bias in individual studies**

| **Reference** | **Sampling** | **Source of infor-mation** | **Recruit-ment years** | **Specification of length of follow-up** | **Study type** | **Overall Risk of Bias** |
| --- | --- | --- | --- | --- | --- | --- |
| **Laparoscopic cholecystectomy** | | | | | | |
| Gundogdu 2017 | - | - | + | + | + | MODERATE |
| Ulrych 2016 | + | + | + | + | + | LOW |
| Pakaneh 2012 | + | + | - | + | + | LOW |
| Ntourakis 2011 | - | + | - | + | + | MODERATE |
| Triantafyllidis 2009 | + | - | - | - | + | HIGH |
| Rathore 2007 | + | - | - | + | + | MODERATE |
| Lindberg 2006 | - | + | - | + | + | MODERATE |
| **Laparoscopic sleeve gastrectomy** | | | | | | |
| Abuoglu 2019 | + | + | + | - | + | LOW |
| AlKhaldi 2019 | + | - | + | + | + | LOW |
| Brunetti 2018 | + | + | + | + | - | LOW |
| Nimeri 2018 | + | + | + | + | + | LOW |
| Thereaux 2018 | + | - | + | + | + | LOW |
| Moradian 2017 | + | - | + | + | + | LOW |
| Sakran 2016 | + | + | - | + | + | LOW |
| Alsina 2014 | + | + | + | + | + | LOW |
| Biertho 2014 | + | + | - | + | + | LOW |
| Woo 2013 | + | + | + | + | + | LOW |
| **Open gastric bypass** | | | | | | |
| Santo 2013 | + | - | - | + | + | MODERATE |
| Slotman 2010 | - | - | - | - | - | HIGH |
| Caruana 2009 | + | - | - | + | + | MODERATE |
| Gargiulo 2007 | + | - | - | + | + | MODERATE |
| Abou-Nukta 2006 | - | - | - | - | + | HIGH |
| Gargiulo 2006 | - | - | - | - | + | HIGH |
| Cotter 2005 | - | - | - | - | - | HIGH |
| **Open groin hernia repair** | | | | | | |
| Poudel 2020 | + | - | + | + | - | MODERATE |
| Wang 2020 | + | - | + | - | + | MODERATE |
| Lozano 2015 | - | + | - | + | + | MODERATE |
| Bessa 2015 | + | + | - | - | + | MODERATE |
| Srsen 2008 | + | - | - | - | + | HIGH |
| Holzheimer 2007 | + | + | - | - | + | MODERATE |
| **Open liver resection** | | | | | | |
| Ainoa 2020 | + | - | + | + | + | LOW |
| Kron 2019 | + | - | - | + | + | MODERATE |
| Singh 2017 | + | - | + | - | + | MODERATE |
| Aramaki 2014 | + | - | - | + | + | MODERATE |
| Nathan 2014 | + | + | - | + | + | LOW |
| Barbas 2013 | + | - | - | + | + | MODERATE |
| **Open radical hysterectomy** | | | | | | |
| Neagoe 2018 | - | - | - | + | - | HIGH |
| Thomas 2017 | + | - | + | + | - | MODERATE |
| Djurdjevic 2015 | + | - | - | - | - | HIGH |
| Gao 2015 | - | - | - | + | - | HIGH |
| Satoh 2013 | - | - | - | - | - | HIGH |
| **Open ovarian cancer surgery** | | | | | | |
| Heus 2021 | + | - | + | + | + | LOW |
| Wagner 2019 | + | - | - | + | - | HIGH |
| Fotopoulou 2016 | + | - | + | + | - | MODERATE |
| Lu 2016 | + | - | - | + | - | HIGH |
| Plotti 2016 | - | + | - | - | + | HIGH |
| Krasinski 2014 | + | + | - | + | - | MODERATE |
| Abu Saadeh 2013 | + | - | - | + | - | HIGH |
| Woelber 2010 | + | - | - | + | - | HIGH |
| Mourton 2005 | + | - | - | + | - | HIGH |
